# Total Synthesis of Chiral Falcarindiol Analogues Using BINOL-Promoted Alkyne Addition to Aldehydes

**DOI:** 10.3390/molecules21010112

**Published:** 2016-01-19

**Authors:** Li Wang, Ping-Ping Shou, Si-Ping Wei, Chun Zhang, Shuang-Xun Li, Ping-Xian Liu, Xi Du, Qin Wang

**Affiliations:** 1 Department of Medicinal Chemistry, Sichuan Medical University, Luzhou 646000, China; liwang_512@163.com (L.W.); sh362135508@163.com (P.-P.S.); swei1225@gmail.com (S.-P.W.); zc83good@126.com (C.Z.); 18183382321@sina.cn (S.-X.L.); bojizihan@sina.cn (P.-X.L.); dx.d@163.com (X.D.); 2 Key Laboratory of Medicinal Electrophysiology of Ministry of Education, Sichuan Medical University, Luzhou 646000, China

**Keywords:** falcarindiol analogues, total synthesis, diynol, BINOL, asymmetric addition, alkyne

## Abstract

An enantioselective total synthesis of chiral falcarindiol analogues from buta-1,3-diyn-1-yltriisopropylsilane is reported. The key step in this synthesis is BINOL-promoted asymmetric diacetylene addition to aldehydes. The two chiral centers of the falcarindiol analogues can be produced by using the same kind of catalyst with high selectivity, and the final product can be obtained in only six steps.

## 1. Introduction

**Figure 1:**
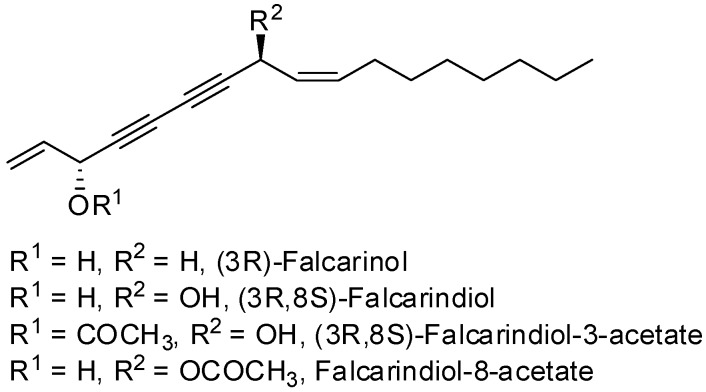
Natural bioactive propargyl alcohols.

Chiral diynol moieties can be found in a number of natural products with a broad array of important biological properties, such as antifungal, antivirus and anticancer properties (Figure 1) [1,2,3]. The unique diynol structure and the configuration of the chiral centers are believed to be closely associated with their biological functions [4]. For example, (3*R*,8*S*)-falcarindiol, which has a typical diynol moiety, has shown great anticancer activity and neuron-protective effect at low concentrations [5,6,7,8]. However, the low content of falcarindiol analogues in natural resources limits their further study and application [9].

Thus total synthesis of the falcarindiol analogues has become an important practical choice in solving this problem, and the construction of the chiral diynol is the key aspect to the synthesis of these natural products or their imitations. Among all of the attempts to construct the chiral diynols, the direct addition of a terminal 1,3-diyne to an aldehyde is the most efficient way. Lots of early works on the enantioselective alkynylation of aldehydes have proved the practicality of this method [10,11,12]. With this approach, the chiral center is formed along with the formation of the carbon-carbon bond; hence, the configuration of the diynols is controllable by using a proper catalyst. A few groups have devoted their work in this aspect and have made impressive progress, though there are still limitations in their methods [13,14,15]. 

Yang *et al.* have synthesized the falcarinol and panaxjapyne A by the asymmetric addition of alkynylzinc reagents to acrolein and propionaldehyde catalyzed by a 1,1’-bi-2-naphthol (BINOL)/Ti(O^i^Pr)_4_ complex and a classic Cadiot-Chodkiewicz cross-coupling reaction with high enantioselectivity (>99% ee) [16]. Zheng *et al.* have successfully synthesized (*S*)-strongylodiol A and (*S*)-strongylodiol B, which were extracted from an Okinawan marine sponge of the genus Strongylophora and exhibited cytotoxic activities against tumor cells, by using a zinc–amino alcohol complex catalyzed 1,3-diynes addition to aldehydes with respective ee values of 55% and 58% [17,18]. However, in these reports, the chiral diynols with two or more chiral centers, such as falcarindiol or panaxytriol, were not investigated. Trost *et al.* employed their dinuclear zinc prophenol catalyst to produce chiral terminal diynols, followed by a regioselective ring-opening of the siloxy epoxide to give the (3*R*,9*R*,10*R*)-panaxytriol in high yield [19]. This efficient catalytic system was also applied in the synthesis of minguartynoic acid, which is a kind of polyacetylene chiral alcohol [20]. Kavirayani R. Prasad finished the total synthesis of natural products panaxytriol (in 12 steps) and (3*S*,10*R*)-panaxydiol (in 11 steps) from the L-tartaric acid derived γ-hydroxy amide [21]. Our previous research discovered that BINOL in combination with ZnEt_2_ and Ti(O^i^Pr)_4_ could catalyze a broad range of alkyne additions to aldehydes with high enantioselectivity [22,23]. This system was greatly improved by the addition of an appropriate Lewis base, such as dicyclohexylamine (Cy_2_NH). Under this condition, 1,3-diynes bearing silyl, aryl, vinyl, and alkyl substituents can react with aromatic or aliphatic aldehydes, especially the α,β-unsaturated aldehydes, to construct 1-subtituted-penta-2,4-diyn-1-ol with high yield and enantioselectivity [24].

In this paper, we wish to report the synthesis of a series of chiral falcarindiol analogues by using the BINOL catalyst system. We have demonstrated that this method is very efficient for generating the final products in short steps with high selectivity, and the two chiral centers within the falcarindiol can be produced by using the same catalyst system.

## 2. Results and Discussion

**Figure 2:**
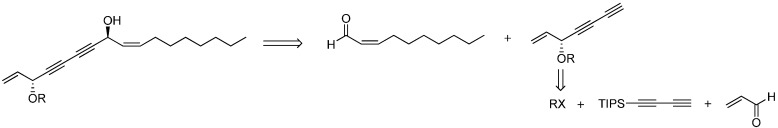
The retrosynthesis for falcarindiol.

Previously, Meng *et al.* reported the purification of natural polyynes and their acetylated products from the root bark of *Oplopanax horridus* (Devil’s Club) and evaluated them as anticancer reagents. Primary structure-activity analysis suggested that the terminal ethylenic bonds of polyacetylenes (Figure 1) would influence their activity while the protection of single hydroxyl group might not influence the inhibitions very much [25,26]. Inspired by these results, we designed the synthetic route of falcarindiol analogues by constructing the first chiral center at the C-8 with an alkyne addition to aldehyde. The hydroxyl group (C-8), which was formed in this step, could be easily protected by an acetyl or benzoyl group, and then the intermediate polyynes with a single chiral center could be delivered into the second alkyne addition step. As depicted in our retrosynthetic analysis of falcarindiol analogues (Figure 2), the main building blocks would be a protected diyne and suitable aldehydes.

**Scheme 1:**
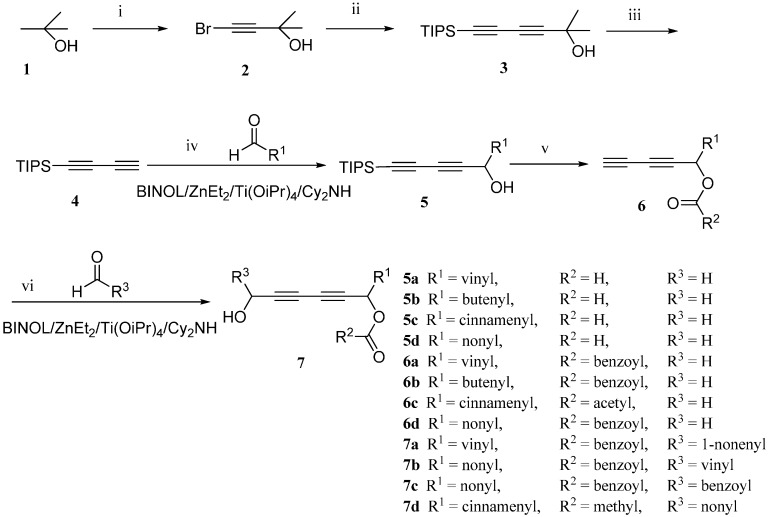
The total synthesis route of chiral falcarindiol analogues. (i) KOH, Br_2_, 0 °C, 0.5 h; then 25 °C, 0.5 h, 90%; (ii) BuNH_2_, CuCl, NH_2_OH·HCl, triisopropylsilylacetylene, 2 h, 0 °C, 89%; (iii) KOH, toluene, reflux, 4 h, 84%; (iv) R-BINOL, Cy_2_NH, ZnEt_2_, Et_2_O, 16 h, room temperature; Ti(O^i^Pr)_4_, 2 h, room temperature; aldehyde, 4 h, room temperature, 80%–99%; (v) DMAP, CH_2_Cl_2_, 0 °C, Et_3_N, anhydride, 0 °C, 2 h; AcOH, TBAF, 0 °C,40 min, 72%–89%; (vi) *R*-BINOL, Cy_2_NH, ZnEt_2_, Et_2_O, room temperature, 16 h; Ti(O^i^Pr)_4_, room temperature, 2 h; aldehyde, room temperature, 4 h, 17%–97%.

The synthetic sequence started from the preparation of the diyne. The intermediate buta-1,3-diyn-1-yltriisopropylsilane (**4**) was synthesized according to the method described by Trost *et al.* with our modification, as shown in Scheme 1 [19]. 2-Methylbut-3-yn-2-ol (**1**) was treated with Br_2_ and aq. KOH to afford 4-bromo-2-methylbut-3-yn-2-ol (**2**) in 90% yield. Then 4-bromo-2-methylbut-3-yn-2-ol (**2**) underwent the Cadiot–Chodkiewicz cross-coupling reaction with triisopropylsilylacetylene in the presence of CuCl and amines to give 2-methyl-6-(triisopropylsilyl)hexa-3,5-diyn-2-ol (**3**). After the removal of the 2-hydroxypropyl-group under basic condition, compound **4** was successfully synthesized. Table 1 shows our screening of the reaction conditions for this step. As shown in Table 1, the solvent used played a very important role in this deprotection step. Toluene was found to be the best solvent with the highest yield and less toxic (entry 5). In contrast to the literature [27], the cleavage of 2-methyl-6-(triisopropylsilyl)hexa-3,5-diyn-2-ol (**3**) to form the buta-1,3-diynyltriisopropylsilane was not significantly affected by the addition of K_3_PO_4_ (entry 4 and 5).

**Table 1:** Screening of the conditions for the 2-hydroxypropyl cleavage.

Entry	Solvent	Base/eq.	Additive/eq.	T/°C	T/h	Yield%
1	benzene	KOH/2.2	NULL	81	4	79
2	THF	KOH/1.0	NULL	25	6	9
3	THF	KOH/2.2	K_3_PO_4_/1.6	66	0.7	NULL
4	toluene	KOH/1.0	K_3_PO_4_/1.0	110	1.5	70
5	toluene	KOH/2.2	NULL	110	4	84

With compound **4** in hand, we moved on to the construction of the 1-substituted-5-(triisopropylsilyl)pent-2,4-diynols containing a single chiral center (Table 2). Compound **4** was separately reacted with acraldehyde, (*E*)-pent-2-enal, cinnamaldehyde and decyl aldehyde in the presence of (*R*)-BINOL/ZnEt_2_/Ti(O^i^Pr)_4_/Cy_2_NH to give compounds **5a** to **5d** in 80%–99% yield (Entry 1 to 4). These compounds were converted to compounds **6** by firstly protecting the hydroxyl group of compounds **5** with an ester moiety and then removing the triisopropylsilyl protecting group. The compounds **5** were treated with DMAP, Et_3_N and appropriate anhydride at 0 °C for 2 h in CH_2_Cl_2_, then the TIPS groups were removed with 1.1 equiv. of acetic acid and 1.2 equiv. of tetrabutylammonium fluoride (TBAF) to give compounds **6** in 72%–89% yields over two steps. The enantioselectivity of the BINOL/ZnEt_2_/Ti(O^i^Pr)_4_/Cy_2_NH catalyzed addition of the diyne **4** to the aldehydes was evaluated by measuring the enantiomeric purity of compounds **6** which gave 81%–89% ee. The best enantioselectivity was achieved in the synthesis of **6a**, which bears a vinyl group. According to our previously work and literature [22,24], using *R*-BINOL as chiral source in this catalytic system will lead to the production of alkynol with an *S* configuration. The NMR spectra and HPLC plots of compounds **6** are provided in the Supplementary Materials.

**Table 2:** Synthesis of the intermediate diynols **5** and the corresponding esters **6**.

Entry	product	Yield%	product	Yield%	ee%
1	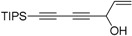 **5a**	91	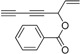 **6a**	72	89
2	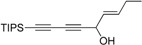 **5b**	87	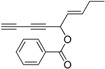 **6b**	86	81
3	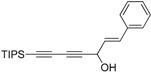 **5c**	80	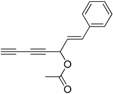 **6c**	72	81
4	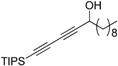 **5d**	99	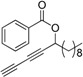 **6d**	89	82

Then we subjected the diynes **6** to the asymmetric addition to aldehydes again by using the same catalyst system ((*R*)-BINOL/ZnEt_2_/Ti(O^i^Pr)_4_/Cy_2_NH) to synthesize a series of falcarindiol analogues. The results are summarized in Table 3. Compound **6a** was reacted with (*Z*)-dec-2-enal to give the 3-OH protected falcarindiol **7a** in 17% yield (Entry 1). Compound **6d** reacted with acraldehyde to give 3-hydroxyheptadeca-1-en-4,6-diyn-8-yl benzoate **7b** (Entry 2), which was a imitate of falcarindiol, in moderate yield (61%) and high selectivity (dr > 99:1). Similarly, **7c** was obtained from the reaction of **6d** with cinnamaldehyde in a high yield of 97% while the diastereoselectivity decreased to 6:1 on the basis of the HPLC analysis (Entry 3). The reaction of **6c** with decyl aldehyde gave **7d** with less than 10% yield, even if the reaction time was prolonged for 25 h. **7d** is not stable and undergoes rapid degradation after the purification. As we applied *R*-BINOL in the catalytic system twice, the configuration of the final product in our experiment, the **7a** for instance, is assigned to be (3*S*,8*S*,*Z*)-8-hydroxyheptadeca-1,9-dien-4,6-diyn-3-yl benzoat. This assignment is also in accordance with the optical rotation data of **7a** [4]. Besides, we realized that the sequence of alkynylation step of aldehydes could influence the purification and the stability of the final products. For example, the **7b** or **7c**, which is produced by alkynylation with decyl aldehyde in the first addition step, has higher yield than those alkynylated with acraldehyde (**7a**) or cinnamaldehyde (**7d**) first. The NMR spectra of compounds **7** were also provided in the Supplementary Materials.

**Table 3:** Synthesis of falcarindiol analogues ^a^.

Entry	Compound	Product	Yield%	dr
1	**7a**	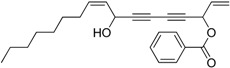	17	14:1 ^b,^^e^
2	**7b**	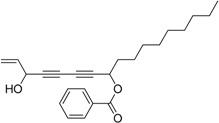	61	>99:1 ^b^
3	**7c**	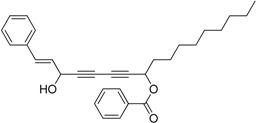	97	6:1 ^c^
4	**7d**	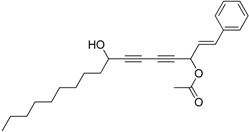	<10 ^d^	-

^a^ The aldehyde was added and stirred for 4 h at room temperature; ^b^ by ^1^H-NMR analysis; ^c^ by HPLC analysis; ^d^ the reaction was quenched after the aldehyde was added and stirred for 24 h; ^e^ ${\left[\alpha \right]}_{D}^{20}$ = 84.1 (*c* = 0.9, in CH_2_Cl_2_).

## 3. Experimental Section

### 3.1. General Methods

All commercial chemicals and solvents were used without further purification. Reactions were monitored by thin layer chromatography (TLC) on silica gel plates (F254) prepared in the laboratory and flash chromatography was performed on silica gel (300–400 mesh). The ^1^H- and ^13^C-NMR spectra were measured on Bruker 400 MHz spectrometer (Bruker, Fällanden, Switzerland) with CDCl_3_ as the solvent. Chemical shifts (ppm) were determined using tetramethylsilane (TMS) as the internal standard and the coupling constants (*J*) are given in hertz. Melting points were determined on a Hanon MP300 melting point measuring device. High-resolution mass spectra were obtained on a Finnigan LCQDECA spectrometer. The optical rotation was measured on a PERKIN ELEMER 341 Polarimeter. The HPLC analysis was using corresponding commercial chiral column (Daicel Chiralcel OD-3 column 250 × 4.6 mm, Daicel Chiralpak AD-H column 250 × 4.6 mm, or Daicel Chiralpak AS-H column 250 × 4.6 mm) as stated in the experimental procedures with UV detection at 254 nm.

### 3.2. Preparation of Buta-1,3-diyn-1-yltriisopropylsilane ***4***

Buta-1,3-diyn-1-yltriisopropylsilane (**4**) was synthesized according to the literature with modifications [12]. Bromine (5 mL, 97.6 mmol, 0.9 eq.) was slowly added to an aq. KOH solution (30 g, 535.7 mmol, 5.0 eq., 200 mL H_2_O) at 0 °C and the stirring was maintained for 30 min. Then 2-methylbut-3-yn-2-ol (10 mL, 108.2 mmol, 1 eq.) was added drop wise to the mixture, which was stirred for 30 min. The reaction mixture was extracted with ether (60 mL × 3) after 40 min standing at room temperature. The organic layers were combined, dried over Na_2_SO_4_, and then evaporated under vacuum. Product **2** was isolated as yellow oil by chromatography (SiO_2_, petroleum ether/AcOEt, 15:1, *v/v*, yield 90%).

CuCl (364.4 mg, 3.7 mmol, 0.02 eq.) was added to a solution of butylamine (154 mL) in water (513 mL). The solution immediately turned blue, and the color changed to orange and then turned to brown by the addition of 0.5 g hydroxylamine hydrochloride. After triisopropylsilylacetylene (40.3 g, 220.8 mmol, 1.2 eq.) was added, the reaction was immediately cooled in an ice bath and the color turned orange. Then the bromoalkyne **2** (30.0 g, 184.0 mmol, 1.0 eq.) was added in slowly, and the reaction was stirred vigorously at 0 °C. Approximately every 2 min 0.5 g H_2_N(OH)·HCl was added, while the solution developed a brown color. The reaction was stirred for additional 2 h and monitored by TLC. The aqueous mixture was extracted with Et_2_O (3 × 50 mL); the combined organic phases were washed with brine (100 mL), and then dried over MgSO_4_. The crude product **3** was purified by column chromatography (petroleum ether:EtOAc = 10:1, yield = 89%) to afford a yellow oil.

A toluene (390 mL) solution of the TIPS protected dynol **3** (2.097 g, 7.9 mmol, 1 eq.) was heated at reflux in the presence of KOH (978.5 mg, 17.4 mmol, 2.2 eq.) for 4 h and then filtered through diatomaceous earth. After removal of the solvent under vacuum, the buta-1,3-diyn-1-yltriisopropylsilane **4** was isolated as a pale yellow oil by column chromatography (SiO_2_, petroleum ether only, yield 89%).

### 3.3. Preparation of 7-(Triisopropylsilyl)hepta-1-en-4,6-diyn-3-ol ***5a***

Under the protection of nitrogen, diethyl zinc (2.4 mL, 2 M in toluene, 4.8 mmol, 3.0 eq.) was slowly added to an ether (20 mL) solution of dicyclohexylamine (15.4 μL, 14.0 mg, 0.08 mmol, 0.05 eq.), *R*-BINOL (183.0 mg, 0.6 mmol, 0.4 eq.) and buta-1,3-diyn-1-yltriisopropylsilane **4** (1.0 g, 4.8 mmol, 3.0 eq.). The mixture was stirred at room temperature for 16 h, and then Ti(O*^i^*Pr)_4_ (474 μL, 1.6 mmol, 1.0 eq.) was slowly added. After additional 2 h, acraldehyde (107 μL, 1.6 mmol, 1.0 eq.) was added and the resulting solution was stirred for 5 h. Then, the reaction was quenched with saturated ammonium chloride (6 mL). The resulting mixture was extracted with methylene chloride (5 mL × 3), dried over sodium sulfate and concentrated under vacuum. The residue was passed through a short silica gel column (petroleum ether/AcOEt, 30:1, *v/v*) to give **5a** as light yellow oil in 91% yield. ^1^H-NMR (400 MHz, CDCl_3_) δ 6.02–5.94 (m, 1H), 5.56–5.46 (m, 1H), 5.32–5.26 (m, 1H), 4.97 (m, 1H), 1.93 (d, *J* = 6.4 Hz, 1H), 1.10 (s, 21H).

### 3.4. Preparation of (E)-1-(Triisopropylsilyl)nona-6-en-1,3-diyn-5-ol ***5b***

Under the protection of nitrogen, buta-1,3-diyn-1-yltriisopropylsilane **4** (742 μL, 3.0 mmol, 1.5 eq.), dicyclohexylamine (19.2 μL, 0.1 mmol, 0.05 eq.) and diethyl zinc (1.5 mL, 2 M in toluene, 3.0 mmol, 1.5 eq.) were sequentially added to an ether (20 mL) solution of *R*-BINOL (228.8 mg, 0.8 mmol, 0.4 eq.) in a 50 mL flask. The mixture was stirred at room temperature for 16 h, and then Ti(O*^i^*Pr)_4_ (592 μL, 2.0 mmol, 1.0 eq.) was slowly added in. After additional 2 h, (*E*)-2-pentenal (196 μL, 2.0 mmol, 1.0 eq.) was added and the reaction mixture was stirred for 4 h. Then the reaction was quenched with the addition of saturated ammonium chloride (8 mL). The resulting mixture was extracted with methylene chloride (5 mL × 3), dried over sodium sulfate and concentrated under vacuum. The residue was passed through a short silica gel column (petroleum ether/AcOEt, 20:1, *v/v*) to give **5b** as light yellow oil in 87% yield. ^1^H-NMR (400 MHz, CDCl_3_) δ 5.94 (m, 1H), 5.58 (m, 1H), 4.89 (m, 1H), 2.16–2.05 (m, 2H), 1.87 (d, *J* = 5.6 Hz, 1H), 1.08 (s, 21H), 1.02 (t, *J* =7.6 Hz, 3H). ^13^C-NMR (100 MHz, CDCl_3_) δ 139.7, 136.6, 126.9, 88.8, 85.5, 75.6, 71.2, 63.5, 25.0, 18.5, 13.0, 11.2. HRMS: calcd for [M + Na^+^, C_18_H_30_NaOSi]^+^ 313.1964, Found 313.1941.

### 3.5. Preparation of (E)-1-Phenyl-7-(triisopropylsilyl)hepta-1-en-4,6-diyn-3-ol ***5c***

Under the protection of nitrogen, buta-1,3-diyn-1-yltriisopropylsilane **4** (1.4 g, 6.6 mmol, 2.0 eq.), dicyclohexylamine (66 μL, 0.3 mmol, 0.1 eq.) and diethyl zinc (8 mL, 2 M in toluene, 16 mmol, 5.0 eq.) were sequentially added to the ether (40 mL) solution of *R*-BINOL (188.8 mg, 0.7 mmol, 0.2 eq.) in a 100 mL flask. The mixture was stirred at room temperature for 24 h, and then Ti(O*^i^*Pr)_4_ (488 μL, 1.6 mmol, 0.5 eq.) was slowly added in. After additional 2 h, cinnamaldehyde (415 μL, 3.3 mmol, 1.0 eq.) was added and the resulting solution was stirred for 12 h. Then the reaction was quenched with the addition of saturated ammonium chloride (15 mL). The resulting mixture was extracted with methylene chloride (10 mL × 3), dried over sodium sulfate and concentrated under vacuum. The residue was passed through a short silica gel column (petroleum ether/AcOEt, 40:1–20:1, *v/v*) to give **5c** as light yellow oil in 87% yield. ^1^H-NMR (400 MHz, CDCl_3_) δ 7.45–7.37 (m, 2H), 7.37–7.30 (m, 2H), 7.28 (m, 1H), 6.76 (d, *J* = 16.0Hz, 1H), 6.27 (dd, *J* = 16.0, 7.2 Hz, 1H), 5.11 (m, 1H), 2.09 (d, *J* =6.4 Hz, 1H), 1.09 (s, 21H).

### 3.6. Preparation of 1-(Triisopropylsilyl)tetradeca-1,3-diyn-5-ol ***5d***

Under the protection of nitrogen, buta-1,3-diyn-1-yltriisopropylsilane **4** (2.6 g, 12.5 mmol, 3.0 eq.), dicyclohexylamine (40 μL, 0.2 mmol, 0.05 eq.) and diethyl zinc (6.3 mL, 12.5 mmol, 2 M in toluene, 3.0 eq.) were sequentially added to an ether (50 mL) solution of *R*-BINOL (477.6 mg, 1.7 mmol, 0.4 eq.) in a 100 mL flask. The mixture was stirred at room temperature for 16 h, and then Ti(O*^i^*Pr)_4_ (1.2 mL, 4.2 mmol, 1.0 eq.) was slowly added in. The reaction was reacted for additional 2 h, after which decyl aldehyde (785 μL, 4.2 mmol, 1.0 eq.) was added and the mixture was stirred for 4 h. Then the reaction was quenched with saturated ammonium chloride (15 mL). The resulting mixture was extracted with methylene chloride (10 mL × 3), dried over sodium sulfate and concentrated under vacuum. The product was isolated by passing the residue through a short silica gel column (petroleum ether/AcOEt, 20:1, *v/v*) to give **5d** as light yellow oil in 99% yield. ^1^H-NMR (400 MHz, CDCl_3_) δ 4.42 (m, 1H), 1.84 (d, *J* =4.8 Hz, 1H), 1.77–1.66 (m, 2H), 1.49–1.39 (m, 2H), 1.28 (m, 12H), 1.12–1.00 (m, 21H), 0.88 (t, *J* = 6.8 Hz, 3H).

### 3.7. Preparation of Hepta-1-en-4,6-diyn-3-yl benzoate ***6a***

DMAP (18.2 mg, 0.15 mmol, 0.1 eq.), benzoic anhydride (363.3 mg, 1.6 mmol, 1.1 eq.), and Et_3_N (220 μL, 1.6 mmol, 1.1 eq.) were sequentially added to a CH_2_Cl_2_ solution (8.0 mL) of **5a** (416.5 mg, 1.5 mmol, 1 eq.) in a 25 mL flask at 0 °C. The reaction mixture was stirred for 2 h, and then quenched by saturated aq. NaHCO_3_ (15 mL). The resulting mixture was extracted with ether (10 mL × 3), and the organic layers were combined and dried with over sodium sulfate. After being concentrated under vacuum, the residue was dissolved in THF (8 mL) and stirred with acetic acid (90 μL, 1.6 mmol, 1.1 eq.) and TBAF (1.8 mL, 1.8 mmol, 1.2 eq. in THF) at 0 °C for 40 min. Then the reaction was quenched with 2 mL water and extracted with ether (10 mL × 3). The organic layers were combined and dried over sodium sulfate. Further purification was achieved by column chromatography (petroleum ether/AcOEt, 20:1, *v/v*) to give **6a** as light yellow oil in 72% yield. ee = 89%, HPLC analysis: Chiralcel OD-3 column, F = 0.9 mL/min, hexane/^i^PrOH = 99.8/0.2, temp = 30 °C, λ = 254 nm, t_1_ = 20.6 min, t_2_ = 22.6 min. ^1^H-NMR (400 MHz, CDCl_3_) δ 8.16–8.01 (m, 2H), 7.69–7.54 (m, 1H), 7.45 (dd, *J* = 10.8, 4.7 Hz, 2H), 6.16 (dd, *J* = 5.6, 0.8 Hz, 1H), 6.00 (ddd, *J* = 16.8, 10.4, 5.6 Hz, 1H), 5.65 (d, *J* = 16.8Hz, 1H), 5.42 (d, *J* = 10.4 Hz, 1H), 2.24 (d, *J* = 0.8 Hz, 1H). ^13^C-NMR (100 MHz, CDCl_3_) δ 165.0, 133.5, 131.9, 129.9, 129.3, 128.5, 120.0, 71.4, 71.2, 69.3, 67.2, 64.7. HRMS: calcd for [M + Na^+^, C_14_H_10_NaO_2_]^+^ 233.0578, found 233.0547.

### 3.8. Preparation of (E)-Nona-6-en-1,3-diyn-5-yl benzoate ***6b***

DMAP (21.8 mg, 0.17 mmol, 0.1 eq.), benzoic anhydride (434.0 mg, 1.9 mmol, 1.1 eq.), and Et_3_N (262.9 μL, 1.9 mmol, 1.1 eq.) were sequentially added to a CH_2_Cl_2_ solution (9.0 mL) of **5b** (506.7 mg, 1.7 mmol, 1 eq.) in a 25 mL flask at 0 °C. The reaction mixture was stirred for 2 h, and then quenched with saturated aq. NaHCO_3_ (15 mL). The resulting mixture was extracted with ether (10 mL × 3), and the organic layers were combined and dried over sodium sulfate. After being concentrated under vacuum, the residue was dissolved in THF (10 mL) and stirred with acetic acid (107 μL, 1.9 mmol, 1.1 eq.) and TBAF (2.1 mL, 2.1 mmol, 1.2 eq. in THF) at 0 °C for 40 min. Then the reaction was quenched with 2 mL water and extracted with ether (20 mL × 3). The organic layers were combined and dried over sodium sulfate. Further purification was achieved by column chromatography (petroleum ether/AcOEt, 40:1–20:1, *v*/*v*) to give **6b** as light yellow oil in 86% yield. ee = 81%, HPLC analysis: Chiralcel AS-H column, F = 0.4 mL/min, hexane = 100%, temp = 30 °C, λ = 254 nm, t_1_ = 26.2 min, t_2_ = 34.4 min. ^1^H-NMR (400 MHz, CDCl_3_) δ 8.06 (m, 2H), 7.57 (m, 1H), 7.44 (m, 2H), 6.19–6.06 (m, 2H), 5.63 (ddt, *J* = 15.2, 6.4, 1.6 Hz, 1H), 2.24 (d, *J* = 0.8 Hz, 1H), 2.20–2.08 (m, 2H), 1.04 (t, *J* = 7.2 Hz, 3H). ^13^C-NMR (100 MHz, CDCl_3_) δ 165.2, 139.3, 133.3, 129.9, 129.6, 128.4, 122.9, 72.3, 70.7, 69.1, 67.3, 64.8, 25.1, 12.8.

### 3.9. Preparation of (E)-1-Phenylhepta-1-en-4,6-diyn-3-yl acetate ***6c***

DMAP (3 mg, 0.024 mmol, 0.1 eq.), acetic anhydride (25 μL, 0.27 mmol, 1.1 eq.), and Et_3_N (37 μL, 0.27 mmol, 1.1 eq.) were sequentially added to a CH_2_Cl_2_ solution (1.4 mL) of **5c** (82.6 mg, 0.24 mmol, 1 eq.) in a 5 mL flask at 0 °C. The reaction mixture was stirred for 2 h, and then quenched by saturated aq. NaHCO_3_ (2 mL). The resulting mixture was extracted with ether (3 mL × 3) and the organic layers were combined and dried over sodium sulfate. After being concentrated under vacuum, the residue was dissolved in THF (1 mL) and stirred with acetic acid (15 μL, 0.27 mmol, 1.1 eq.) and TBAF (300 μL, 3 mmol, 1.2 eq. in THF) at 0 °C for 1 h. Then the reaction was quenched by 2 mL water and extracted with ether. The organic layers were combined and dried over sodium sulfate. Further purification was achieved by column chromatography (petroleum ether/AcOEt, 50:1–30:1, *v/v*) to give **6c** as light yellow oil in 72% yield. ee = 81%. HPLC analysis: Chiralpak AD-H column, F = 0.8 mL/min, hexane/iPrOH = 95.0/5.0, temp = 30 °C, λ = 254 nm, t_1_ = 9.1 min, t_2_ = 9.7 min. ^1^H-NMR (400 MHz, CDCl_3_) δ 7.46–7.38 (m, 2H), 7.38–7.28 (m, 3H), 6.84 (d, *J* = 15.8 Hz, 1H), 6.20 (dd, *J* = 15.8, 6.8 Hz, 1H), 6.07 (m, 1H), 2.26 (d, *J* = 0.8 Hz, 1H), 2.13 (s, 3H)

### 3.10. Preparation of Tetradeca-1,3-diyn-5-yl benzoate ***6d***

DMAP (53 mg, 0.4 mmol, 0.1 eq.), benzoic anhydride (1.1 g, 5.1 mmol, 1.2 eq.), and Et_3_N (639 μL, 4.7 mmol, 1.1 eq.) were sequentially added to a CH_2_Cl_2_ solution (25.0 mL) of **5d** (1.538 g, 4.2 mmol, 1 eq.) in a 50 mL flask at 0 °C. The reaction mixture was stirred for 2 h, and then quenched with saturated aq. NaHCO_3_ (20 mL). The resulting mixture was extracted with ether (20 mL × 3) and the organic layers were combined and dried over sodium sulfate. After being concentrated under vacuum, the residue was dissolved in THF (25 mL) and stirred with acetic acid (260 μL, 4.7 mmol, 1.1 eq.) and TBAF (4.7 mL, 4.7 mmol, 1.2 eq. in THF) at 0 °C for 40 min. Then the reaction was quenched with 8 mL water and extracted with ether. The organic layers were combined and dried over sodium sulfate. Further purification was achieved by column chromatography (petroleum ether/AcOEt, 80:1–20:1, *v/v*) to give **6d** as light yellow oil in 82% yield. ee = 82%, HPLC analysis: Chiralpak AS-H column, F = 0.8 mL/min, hexane 100%, temp = 30 °C, λ = 254, t_1_ = 8.6 min, t_2_ = 9.6 min. ^1^H-NMR (400 MHz, CDCl_3_) δ 8.09–8.01 (m, 2H), 7.58 (t, *J* = 7.4 Hz, 1H), 7.45 (t, *J* = 7.7 Hz, 2H), 5.63 (t, *J* = 6.6 Hz, 1H), 2.19 (s, 1H), 1.93 (m, 2H), 1.57–1.46 (m, 2H), 1.35–1.23 (m, 12H), 0.88 (t, *J* = 6.8 Hz, 3H). ^13^C-NMR (100 MHz, CDCl_3_) δ 165.4, 133.3, 129.8, 129.6, 128.4, 73.8 69.6, 68.6, 67.4, 64.5, 34.6, 31.9, 29.5, 29.40, 29.3, 29.1, 25.0, 22.7, 14.1. HRMS: calcd for [C_21_H_26_NaO_2_]^+^ 333.1830, Found 333.1836.

### 3.11. Preparation of (Z)-8-Hydroxyheptadeca-1,9-dien-4,6-diyn-3-yl benzoate ***7a***

Under the protection of nitrogen, hepta-1-en-4,6-diyn-3-yl benzoate **6a** (1.1 mmol, 3.0 eq.), dicyclohexylamine (3.4 μL, 0.017 mmol, 0.05 eq.) and diethyl zinc (0.5 mL, 2 M in toluene, 1.1 mmol, 3.0 eq.) were sequentially added to an ether (5 mL) solution of *R*-BINOL (40.0 mg, 0.14 mmol, 0.4 eq.) in a well-dried 10mL flask. The mixture was stirred at room temperature for 16 h, and then a Ti(O*^i^*Pr)_4_ solution (103.6 μL, 0.35 mmol, 1.0 eq.) was slowly added in. After additional 2 h, (*Z*)-dec-2-enal (54.0 μL, 0.35 mmol, 1.0 eq.) was added and the mixture was stirred for 4 h at room temperature. The reaction was quenched with saturated ammonium chloride (5 mL). The resulting mixture was extracted with methylene chloride (5 mL × 3), dried over sodium sulfate and concentrated under vacuum. Final product was obtained by purification with column chromatograph (petroleum ether/AcOEt, 10:1, *v/v*) to give **7a** as light yellow oil in 17% yield. ${\left[\alpha \right]}_{D}^{20}$ = 84.1 (*c* = 0.9, in CH_2_Cl_2_). dr = 14:1. ^1^H-NMR (400 MHz, CDCl_3_) δ 8.07 (m, 2H), 7.58 (m, 1H), 7.45 (m, 2H), 6.18 (d, *J* = 5.6 Hz, 1H), 6.00 (m, 1H), 5.95–5.84 (m, 1H), 5.64 (d, *J* = 17.2 Hz, 1H), 5.56 (dd, *J* = 17.2, 6.0 Hz, 1H), 5.41 (d, *J* = 10.0 Hz, 1H), 4.89 (t, *J* = 6.0 Hz, 1H), 2.05 (m, 2H), 1.93 (m, 1H), 1.37 (m, 2H), 1.29 (m, 8H), 0.88 (t, *J* = 6.8 Hz, 3H). ^13^C-NMR (100 MHz, CDCl_3_) δ 165.1, 135.4, 133.4, 132.0, 129.9, 129.4, 128.5, 127.5, 119.8, 79.5, 74.9, 71.0, 69.6, 65.0, 63.3, 32.0, 31.8, 29.1, 29.1, 28.8, 22.7, 14.1. HRMS [C_24_H_28_NaO_3_]^+^ calcd for 387.1936, Found 387.1923.

### 3.12. Preparation of 3-Hydroxyheptadeca-1-en-4,6-diyn-8-yl benzoate ***7b***

Under the protection of nitrogen, tetradeca-1,3-diyn-5-yl benzoate **6d** (192 μL, 0.6 mmol, 3.0 eq.), dicyclohexylamine (2.0 μL, 0.01 mmol, 0.05 eq.) and diethyl zinc (0.3 mL, 2 M in toluene, 0.6 mmol, 3.0 eq.) were sequentially added to an ether (4 mL) solution of *R*-BINOL (22.9 mg, 0.08 mmol, 0.4 eq.) in a well-dried 10 mL flask. The mixture was stirred at room temperature for 16 h, and then a Ti(O*^i^*Pr)_4_ solution (59 μL, 0.2 mmol, 1.0 eq.) was slowly added in. After additional 2 h, acraldehyde (13.4 μL, 0.2 mmol, 1.0 eq.) was added and the mixture was stirred for 4 h at room temperature. The reaction was quenched with saturated ammonium chloride (5 mL). The resulting mixture was extracted with methylene chloride (5 mL × 3), dried over sodium sulfate and concentrated under vacuum. The final product was obtained by purification with column chromatograph (petroleum ether/AcOEt, 10:1, V/V) which gave **7b** as light yellow oil in 61% yield. dr > 99:1. ^1^H-NMR (400 MHz, CDCl_3_) δ 8.05 (m, 2H), 7.63–7.53 (m, 1H), 7.45 (m, 2H), 5.93 (m, 1H), 5.65 (t, *J* = 6.4 Hz, 1H), 5.47 (d, *J* = 17.2 Hz, 1H), 5.25 (d, *J* = 10.0 Hz, 1H), 4.93 (t, *J* = 5.6 Hz, 1H), 2.16 (d, *J* = 6.0 Hz, 1H), 1.98–1.85 (m, 2H), 1.57–1.46 (m, 2H), 1.35–1.24 (m, 12H), 0.88 (t, *J* = 6.8 Hz, 3H). ^13^C-NMR (100 MHz, CDCl_3_) δ 165.4, 135.7, 133.3, 129.8, 129.6, 128.4, 117.4, 78.1, 77.5, 70.2, 69.3, 64.7, 63.5, 34.6, 31.9, 29.5, 29.4, 29.3, 29.1, 25.0, 22.7, 14.1. HRMS [C_24_H_30_NaO_3_]^+^ calcd for 389.2093, Found 389.2091.

### 3.13. Preparation of (E)-3-Hydroxy-1-phenylheptadeca-1-en-4,6-diyn-8-yl benzoate ***7c***

Under the protection of nitrogen, tetradeca-1,3-diyn-5-yl benzoate **6d** (192 μL, 0.6 mmol, 3.0 eq.), dicyclohexylamine (2.0 μL, 0.01 mmol, 0.05 eq.) and diethyl zinc (0.3 mL, 2 M in toluene, 0.6 mmol, 3.0 eq.) were sequentially added to an ether (4 mL) solution of *R*-BINOL (22.9 mg, 0.08 mmol, 0.4 eq.) in a well-dried 10 mL flask. The mixture was stirred at room temperature for 16 h, and Ti(O*^i^*Pr)_4_ (59 μL, 0.2 mmol, 1.0 eq.) was slowly added in. After additional 2 h, cinnamaldehyde (25.2 μL, 0.2 mmol, 1.0 eq.) was added and the mixture was stirred for 4 h at room temperature. The reaction was quenched with saturated ammonium chloride (5 mL). The resulting mixture was extracted with methylene chloride (5 mL × 3), dried over sodium sulfate and concentrated under vacuum. The final product was obtained by purification with column chromatograph (petroleum ether/AcOEt, 10:1, *v*/*v*) which gave **7c** as light yellow oil in 97% yield. dr = 6:1. HPLC analysis: Chiralcel OD-3 column, F = 0.8 mL/min, hexane/*^i^*PrOH = 94:6, T = 30 °C, λ = 254 nm, t_1_ = 51.8 min, t_2_ = 53.2 min, t_3_ = 58.3 min, t_4_ = 62.2 min. ^1^H-NMR (400 MHz, CDCl_3_) δ 8.05 (dt, *J* = 6.8, 0.8 Hz, 2H), 7.63–7.53 (m, 1H), 7.45 (m, 2H), 7.42–7.36 (m, 2H, 7.36–7.30 (m, 2H), 7.30–7.26 (m, 1H), 6.76 (d, *J* = 16.0 Hz, 1H), 6.25 (dd, *J* = 16.0, 6.0 Hz, 1H), 5.66 (t, *J* = 6.4 Hz, 1H), 5.11 (t, *J* = 6.0Hz, 1H), 2.09–2.01 (m, 1H), 1.93 (m, 2H), 1.52 (m, 2H), 1.40–1.22 (m, 12H), 0.88 (t, *J* = 6.8 Hz, 3H). ^13^C-NMR (100 MHz, CDCl_3_) δ 165.4, 135.8, 133.3, 132.8, 129.8, 129.6, 128.6, 128.4, 128.3, 126.9, 126.7, 78.1, 77.8, 70.4, 69.3, 64.7, 63.3, 34.6, 31.9, 29.5, 29.4, 29.3, 29.1, 25.0, 22.7, 14.1. HRMS [C_30_H_34_NaO_3_]^+^ calcd for 465.2406, Found 465.2391.

### 3.14. Preparation of (E)-8-hydroxy-1-phenylheptadeca-1-en-4,6-diyn-3-yl acetate ***7d***

Under the protection of nitrogen, (*E*)-1-phenylhepta-1-en-4,6-diyn-3-yl acetate **6c** (203.3 mg, 0.9 mmol, 4.0 eq.), dicyclohexylamine (4.4 μL, 0.022 mmol, 0.1 eq.) and diethyl zinc (0.91 mL, 1 M in hexane, 0.91 mmol, 4.0 eq.) were sequentially added to an ether (5 mL) solution of *R*-BINOL (26.3 mg, 0.1 mmol, 0.4 eq.) in a well-dried 10 mL flask at 0 °C. The mixture was stirred at 0 °C for 16 h, and then Ti(O*^i^*Pr)_4_ (68 μL, 0.35 mmol, 1.5 eq.) was slowly added in. After additional 3 h, decanal (42 μL, 0.22 mmol, 1.0 eq.) was added and the mixture was stirred for 24 h. The reaction was quenched with saturated ammonium chloride (5 mL). The resulting mixture was extracted with methylene chloride (5 mL × 3), dried over sodium sulfate and concentrated under vacuum. The final product **7d** was obtained by purification with column chromotograph in less 10% yield with rapid degradation. ^1^H-NMR (400 MHz, CDCl_3_) δ 7.37 (m, 5H), 6.41 (dd, *J* =15.8, 6.4Hz, 1H), 6.32 (d, *J* = 6.4 Hz, 1H), 5.78 (d, *J* = 15.8 Hz, 1H), 4.47 (t, *J* = 6.4 Hz, 1H), 2.13 (s, 3H), 1.89 (s, 1H), 1.78–1.70 (m, 2H), 1.45 (m, 2H), 1.29 (m, 12H), 0.90 (t, *J* = 6.8 Hz, 3H). ^13^C-NMR (100 MHz, CDCl_3_) δ 169.7, 144.0, 137.6, 128.8, 128.6, 127.3, 110.7, 83.8, 76.1, 75.0, 74.9, 69.3, 63.0, 37.5, 31.9, 29.5, 29.5, 29.3, 29.2, 25.0, 22.7, 21.1, 14.1.

## 4. Conclusions

In this study, we have completed the stereoselective synthesis of a series of chiral falcarindiol analogues from buta-1,3-diyn-1-yltriisopropylsilane. The key step in this synthesis is BINOL-promoted asymmetric diacetylene addition to aldehydes. With this method, the two chiral centers of the falcarindiol analogues can be formed by using the same catalyst with high stereoselectivity and the final products can be obtained in short synthetic steps. The evaluation of the anti-proliferative activity of these chiral falcarindiol analogues is undergoing.

## Supplementary Materials

Supplementary materials can be accessed at: http://www.mdpi.com/1420-3049/21/1/112/s1.
