# Supplementary material for: Total Synthesis of Chiral Falcarindiol Analogues Using BINOL-Promoted Alkyne Addition to Aldehydes
Source: Molecules. 2016 Jan 19;21(1):112. doi: 10.3390/molecules21010112 (PMC6274458; doi:10.3390/molecules21010112)
Supplement: Supplementary file 1 [file molecules-21-00112-s001.pdf]

Supplementary Materials: Total Synthesis of Chiral Falcarindiol Analogues Using BINOL-Promoted Alkyne Addition to Aldehydes

Li Wang [1,†], Ping-Ping Shou [1,†], Si-Ping Wei [1], Chun Zhang [1], Shuang-Xun Li [1], Ping-Xian Liu [1], Xi Du [1] and Qin Wang [1,2,*]

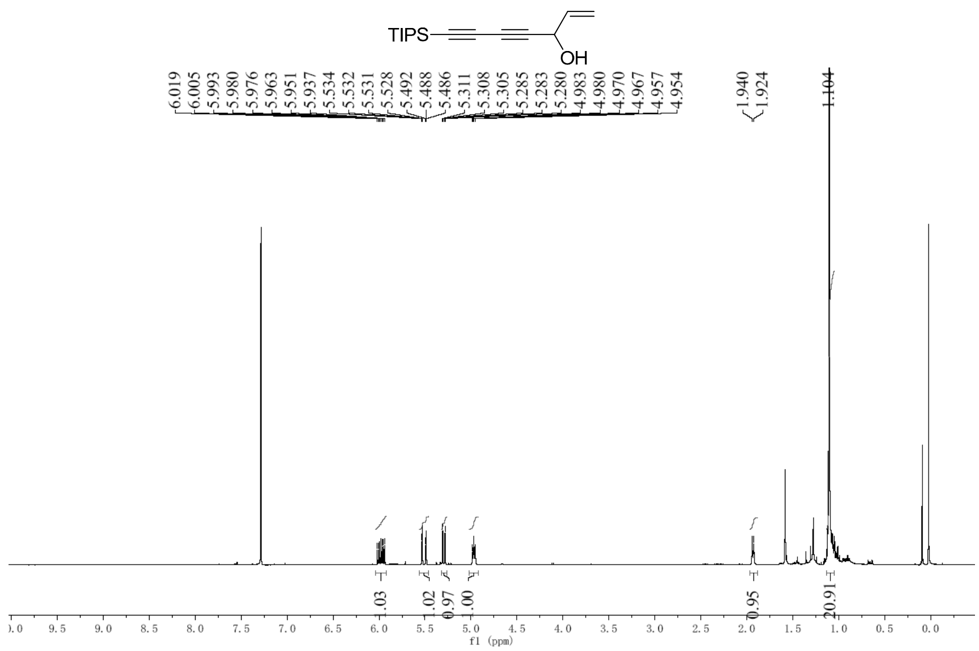

Figure S1. ^{1}H-NMR spectra of compound **5a**.

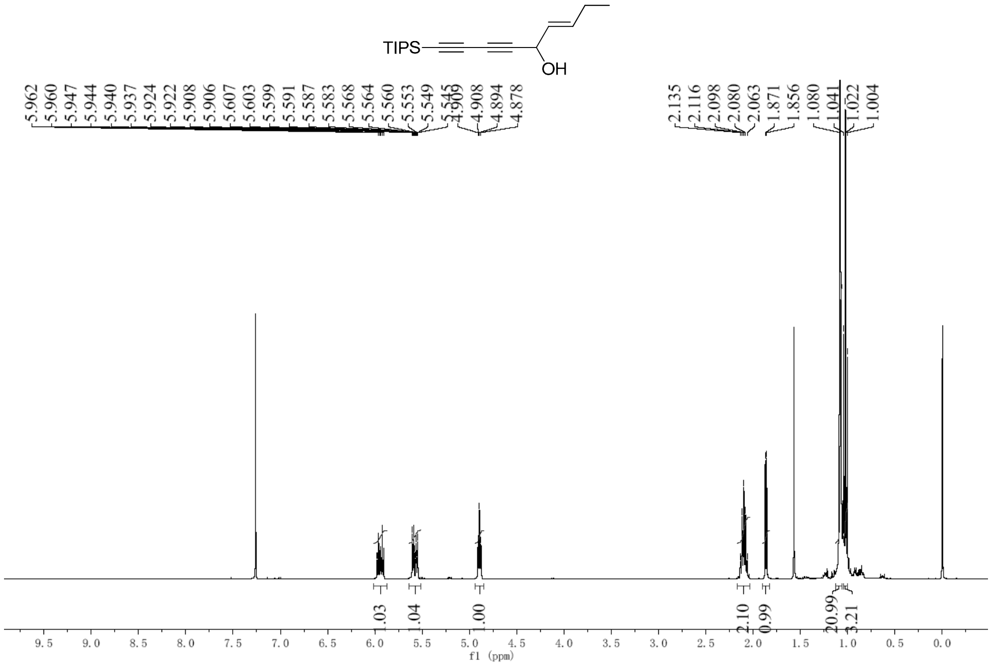

Figure S2. ^{1}H-NMR spectra of compound **5b**.

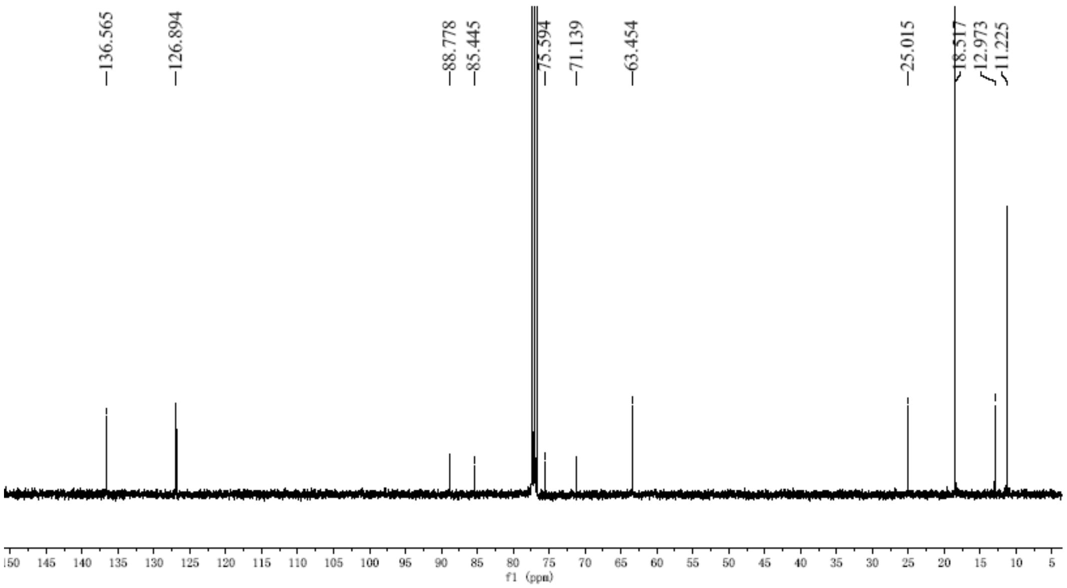

Figure S3. ^{13}C-NMR spectra of compound **5b**.

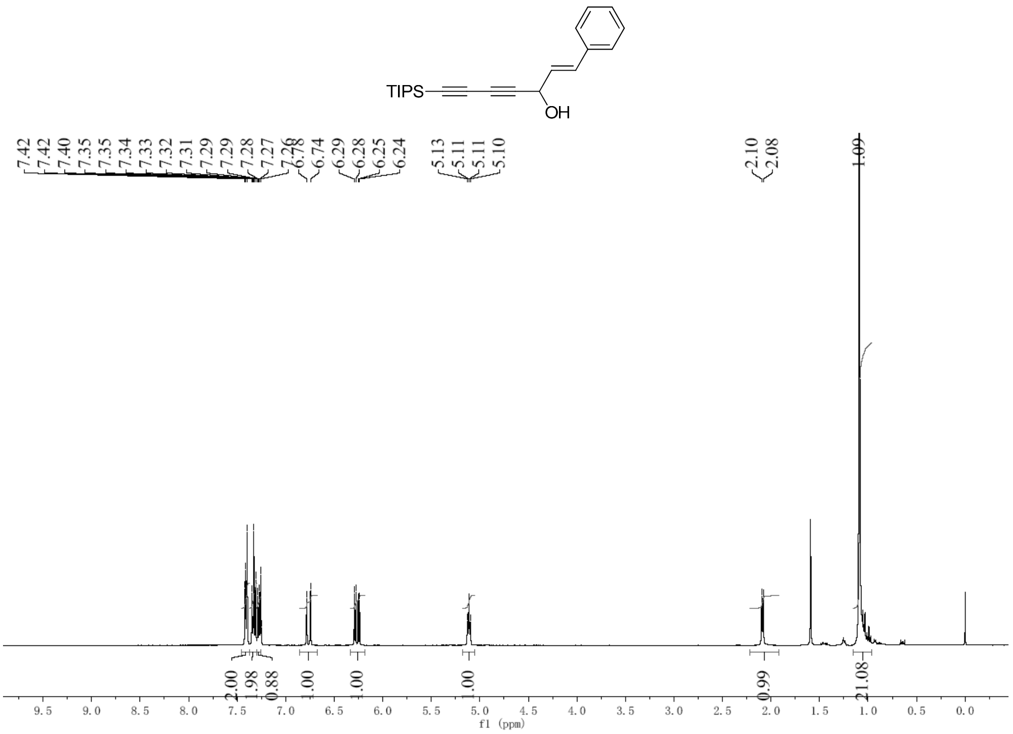

Figure S4. ^{1}H-NMR spectra of compound **5c**.

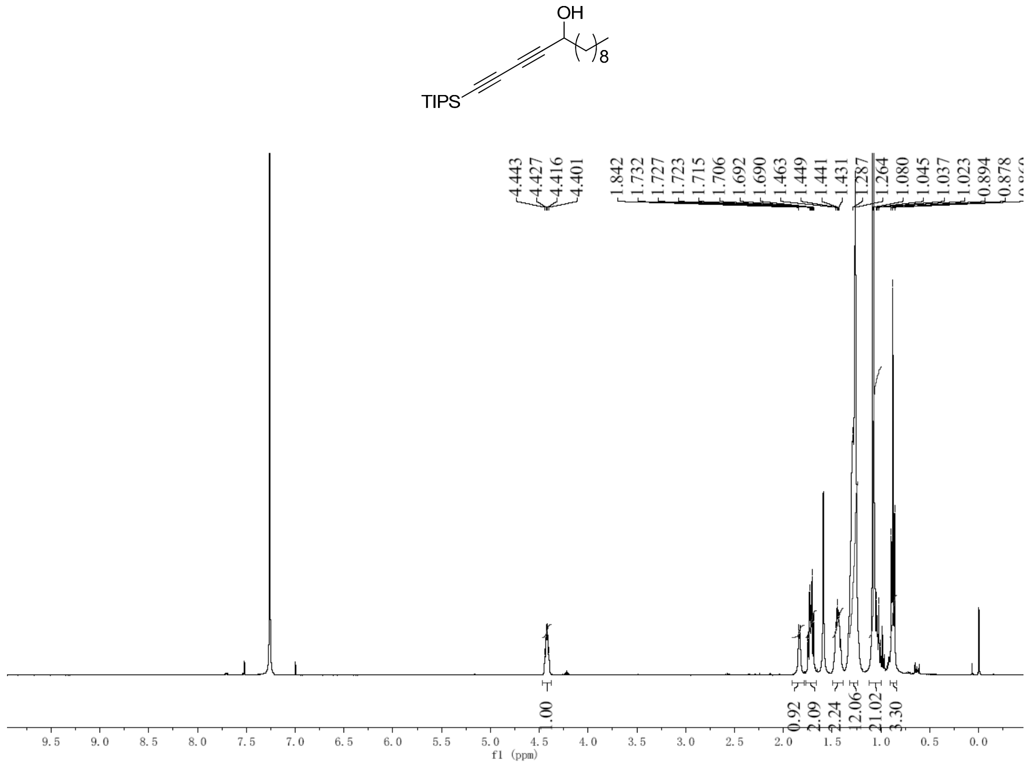

Figure S5. ^{1}H-NMR spectra of compound **5d**.

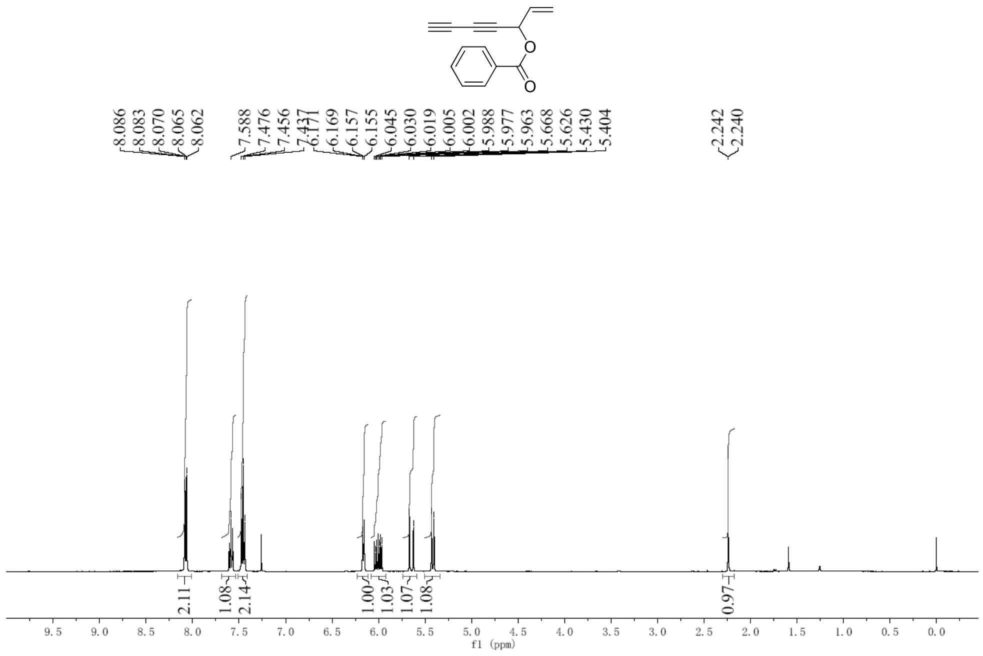

Figure S6. ^{1}H-NMR spectra of compound **6a**.

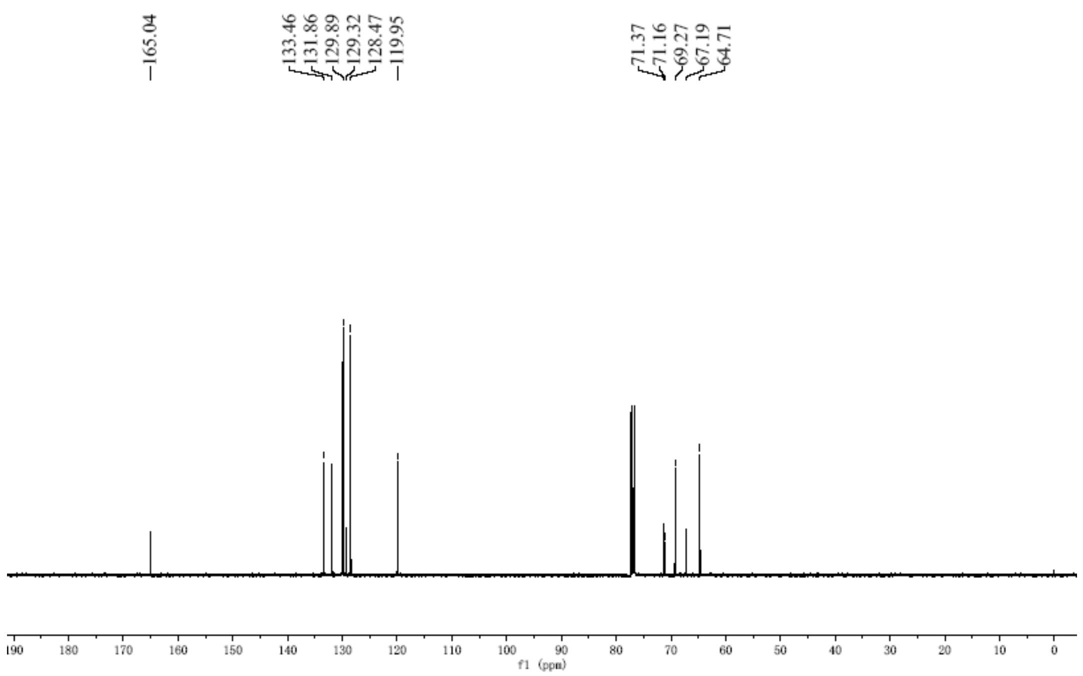

Figure S7. ^{13}C-NMR spectra of compound **6a**.

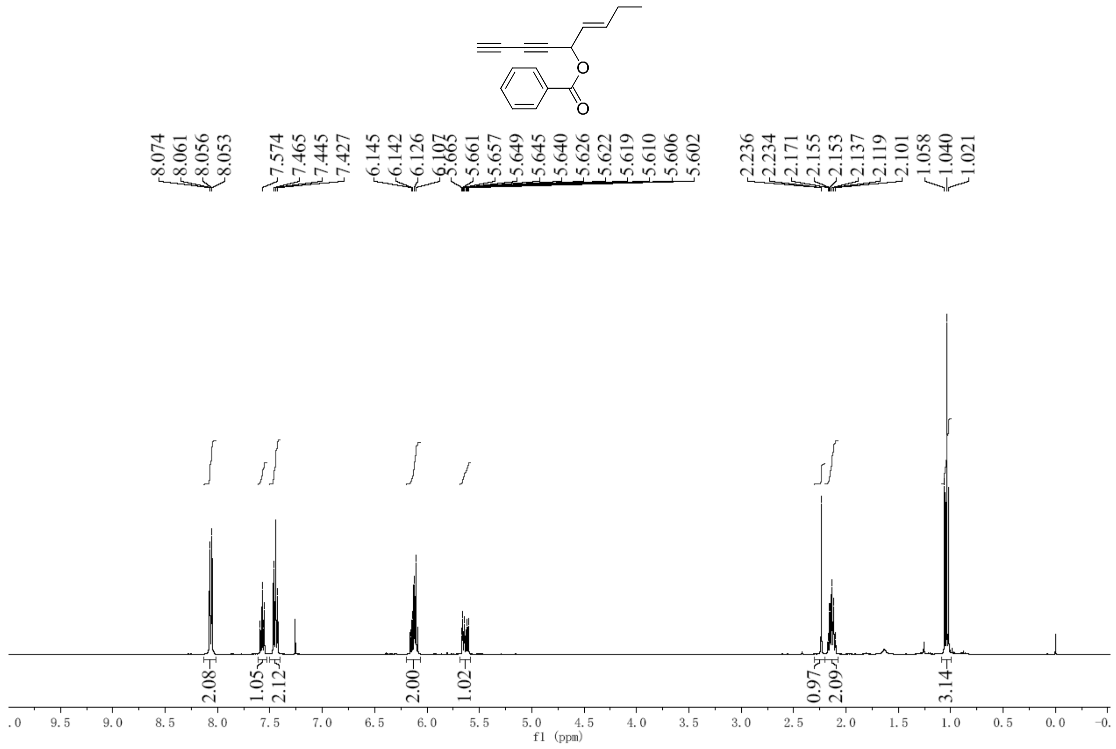

Figure S8. ^{1}H-NMR spectra of compound **6b**.

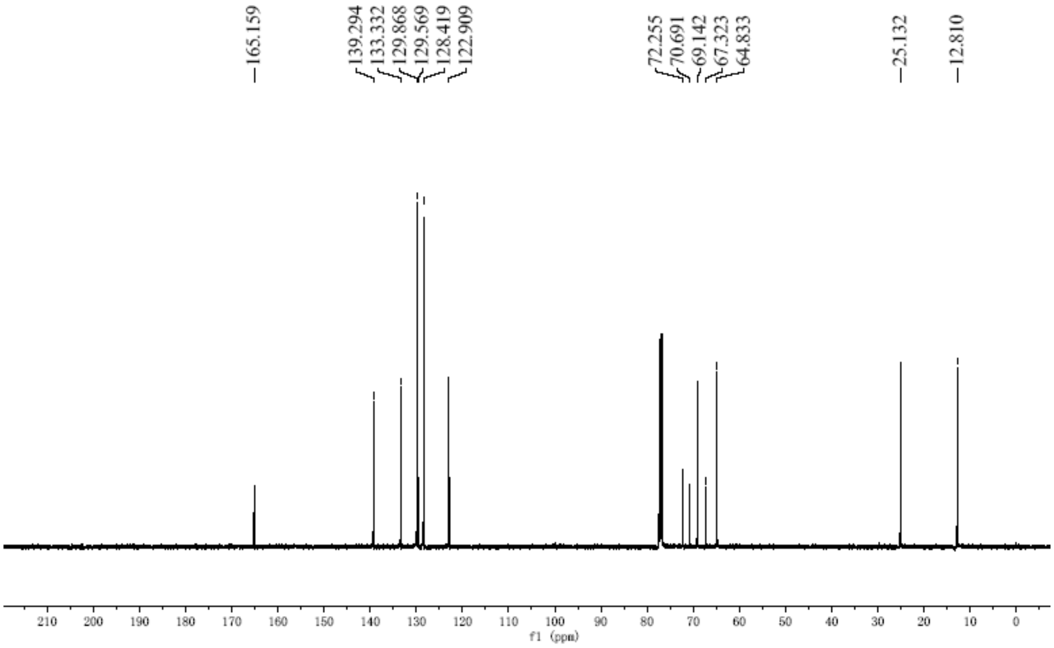

Figure S9. ^{13}C-NMR spectra of compound **6b**.

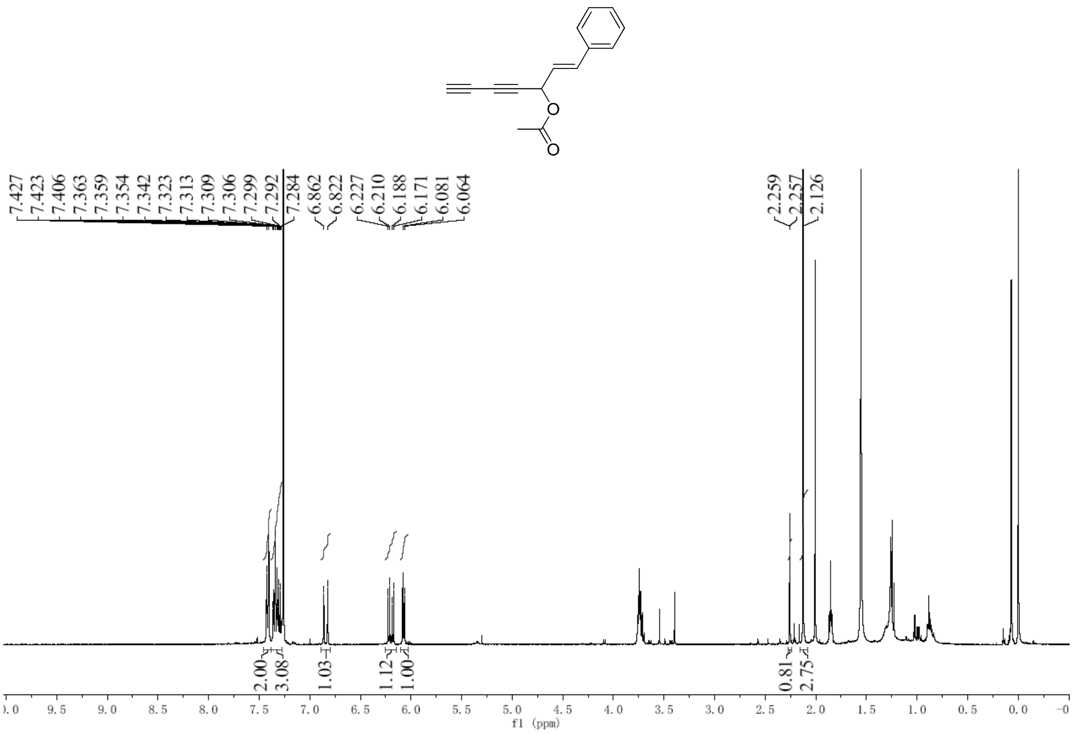

Figure S10. ^{1}H-NMR spectra of compound **6c**.

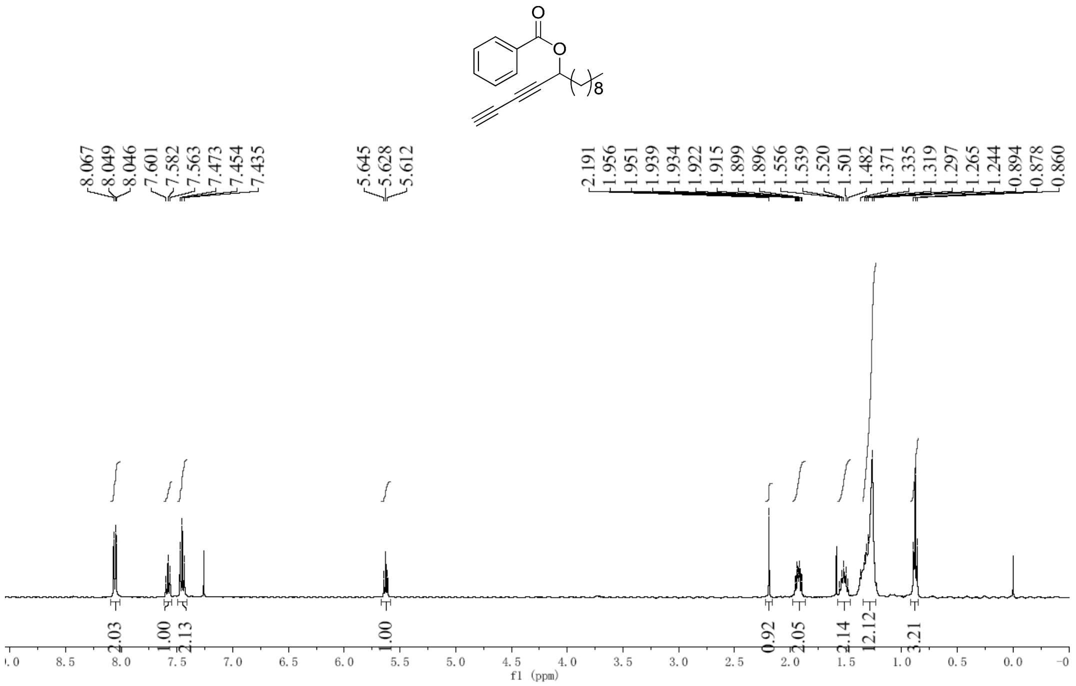

Figure S11. ^{1}H-NMR spectra of compound **6d**.

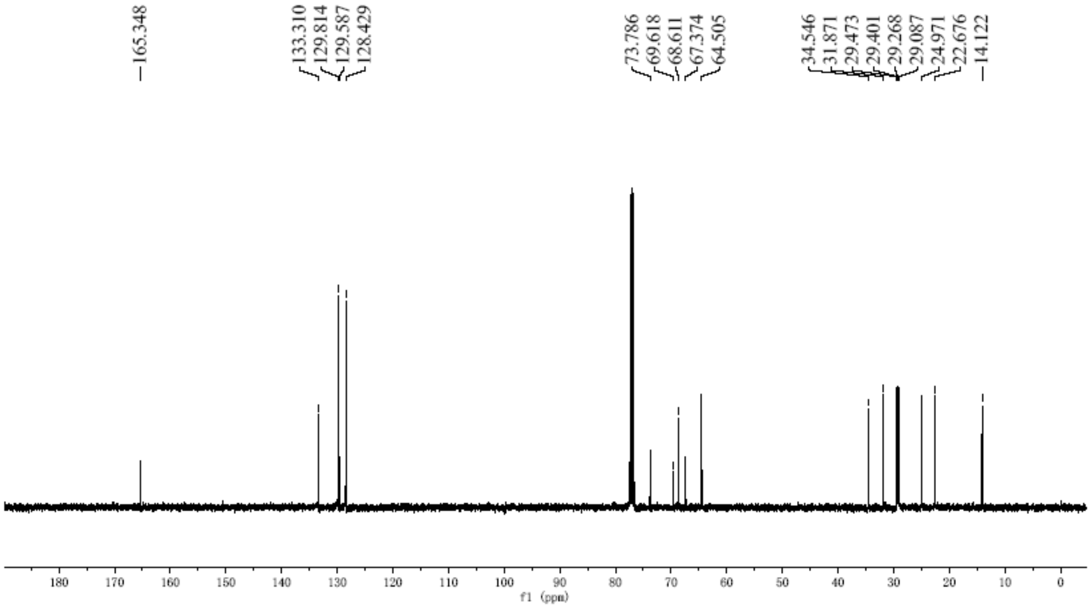

Figure S12. ^{13}C-NMR spectra of compound **6d**.

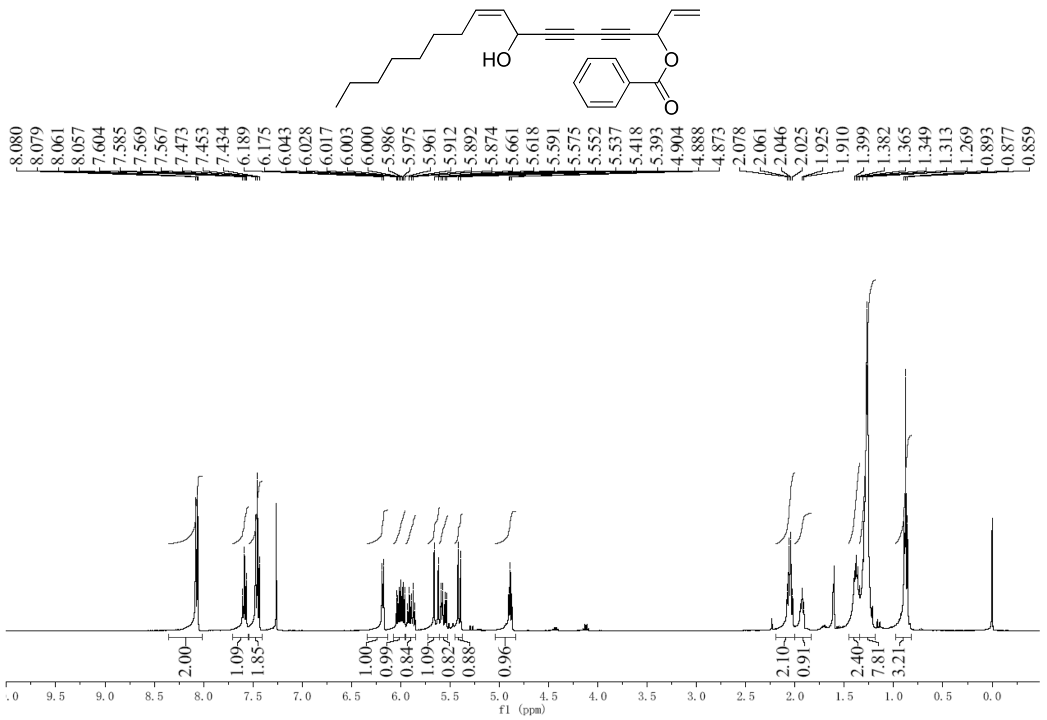

Figure S13. ^{1}H-NMR spectra of compound **7a**.

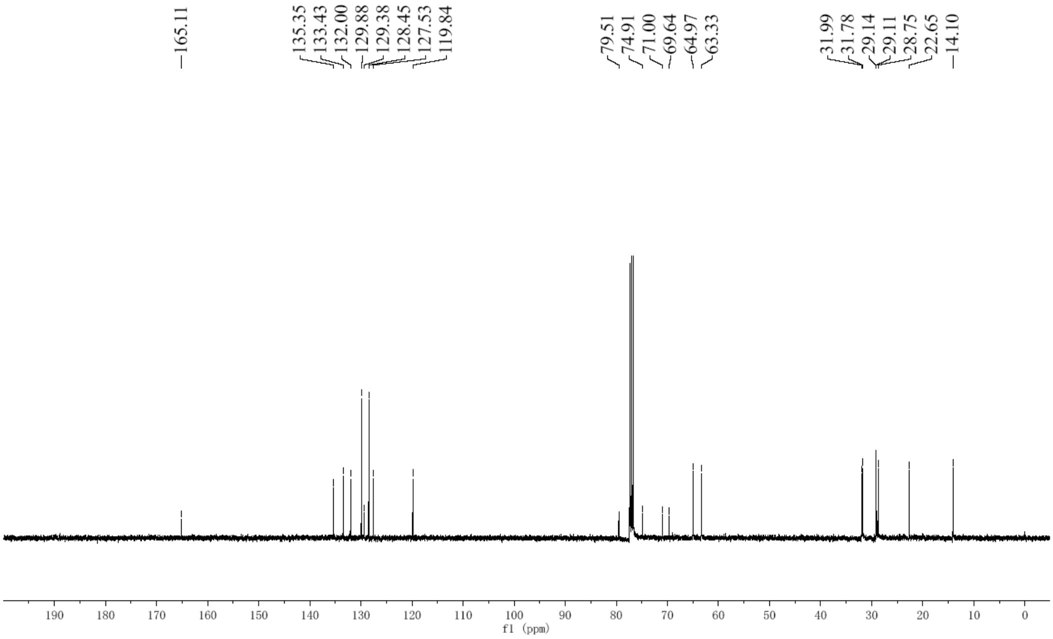

Figure S14. ^{13}C-NMR spectra of compound **7a**.

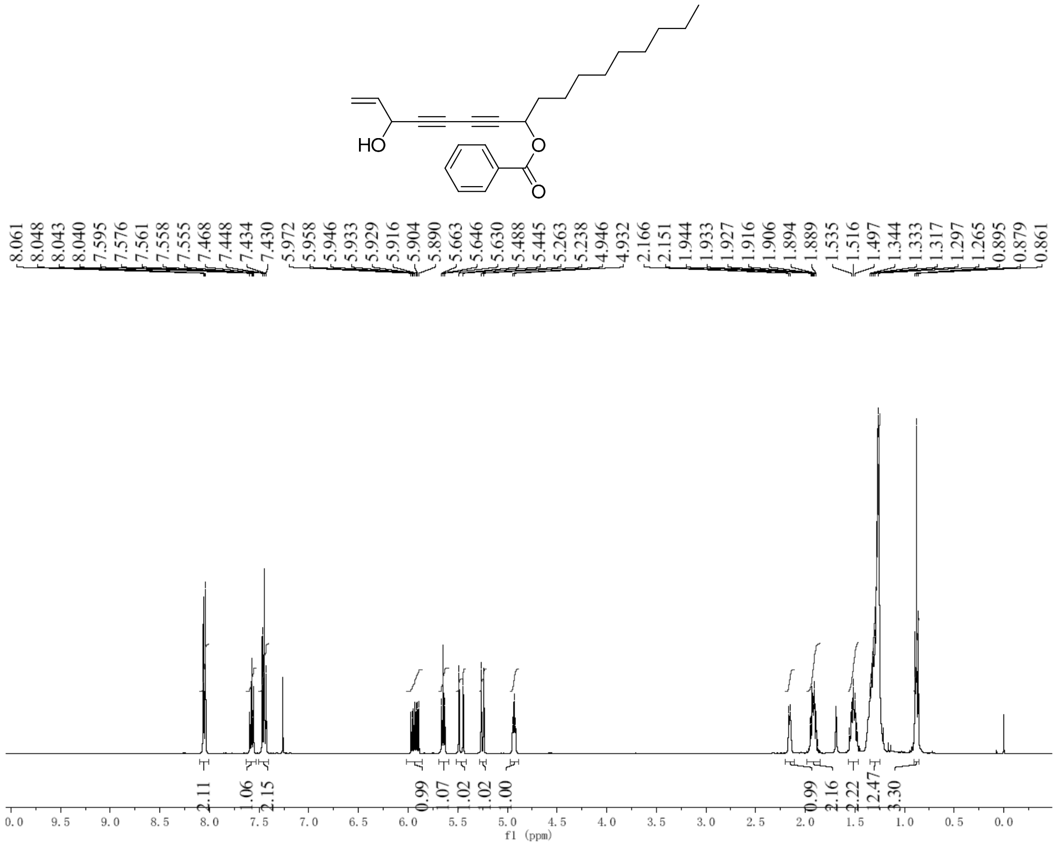

Figure S15. ^{1}H-NMR spectra of compound **7b**.

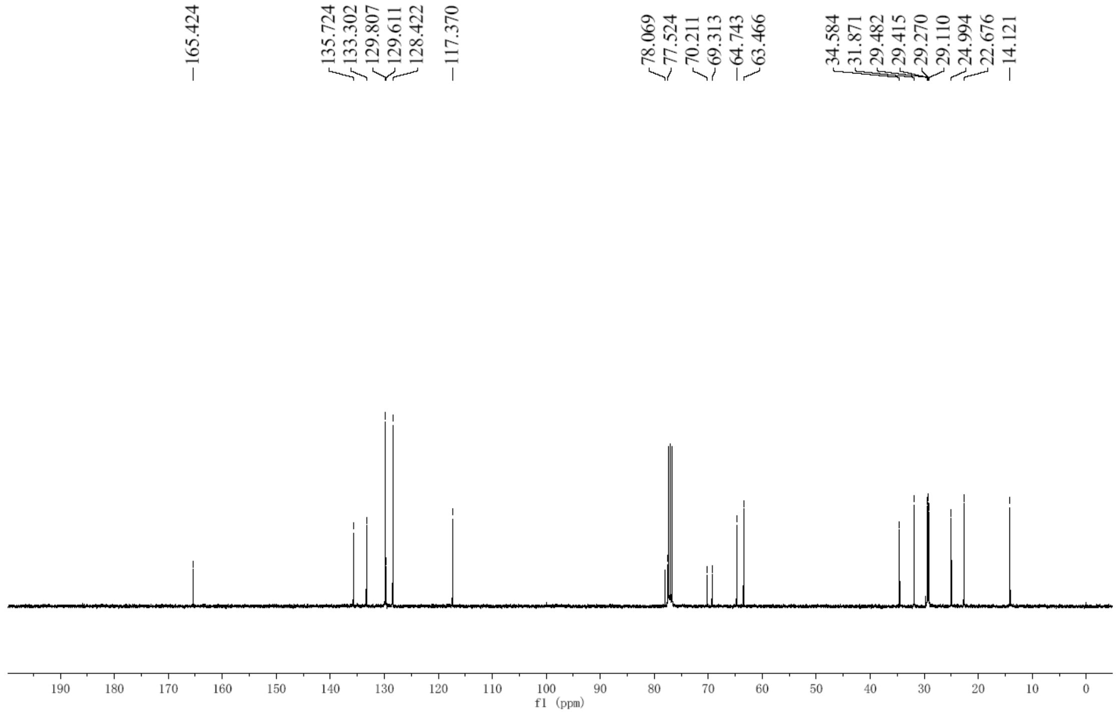

Figure S16. ^{13}C-NMR spectra of compound **7b**.

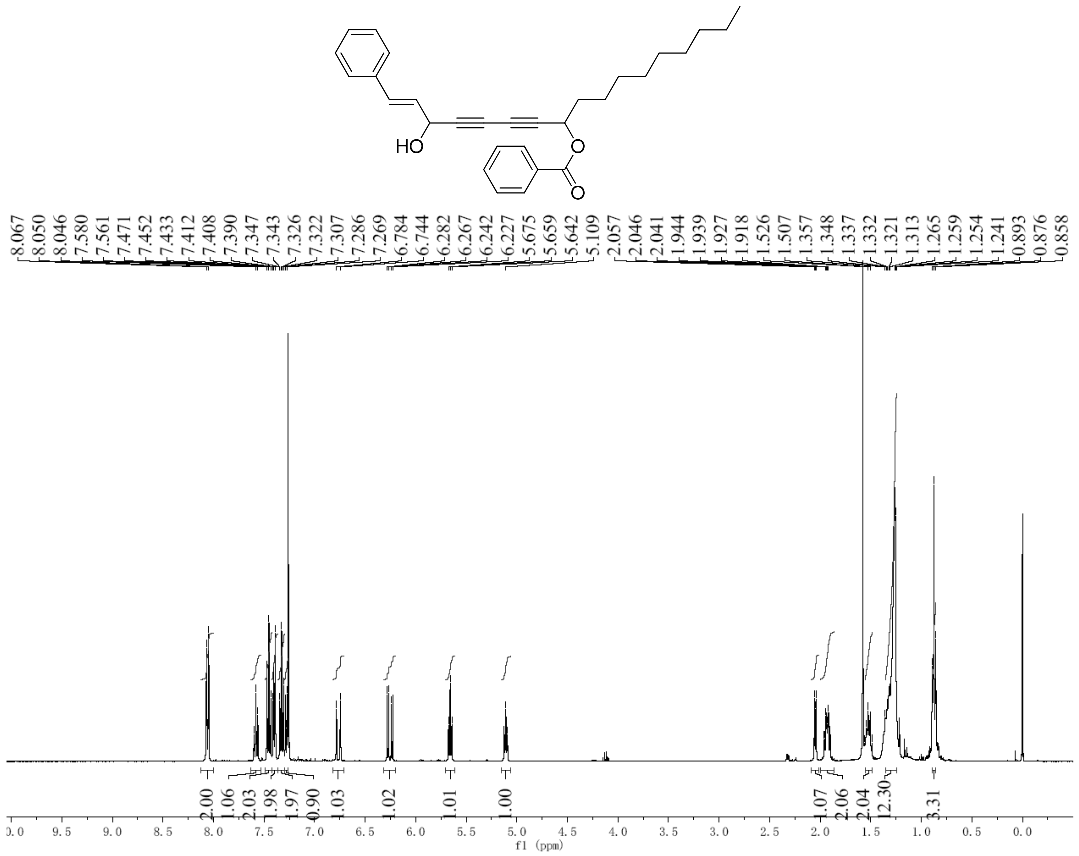

Figure S17. ^{1}H-NMR spectra of compound **7c**.

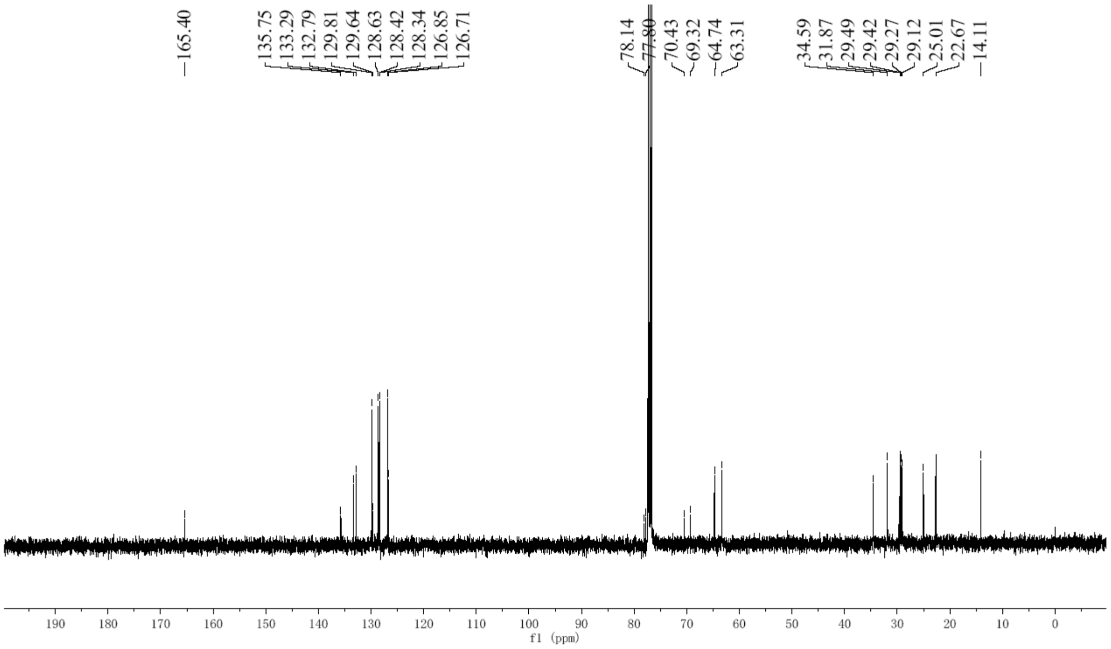

Figure S18. ^{13}C-NMR spectra of compound **7c**.

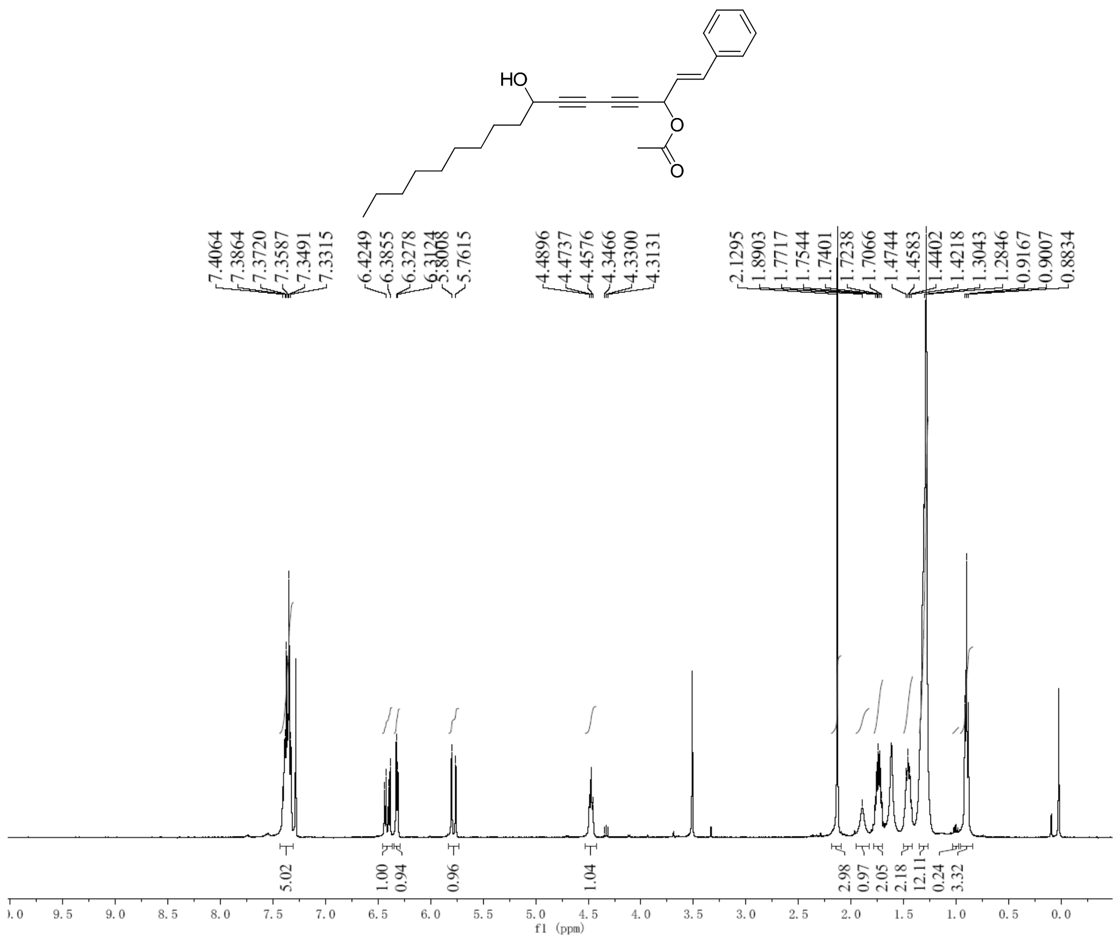

Figure S19. ^{1}H-NMR spectra of compound **7d**.

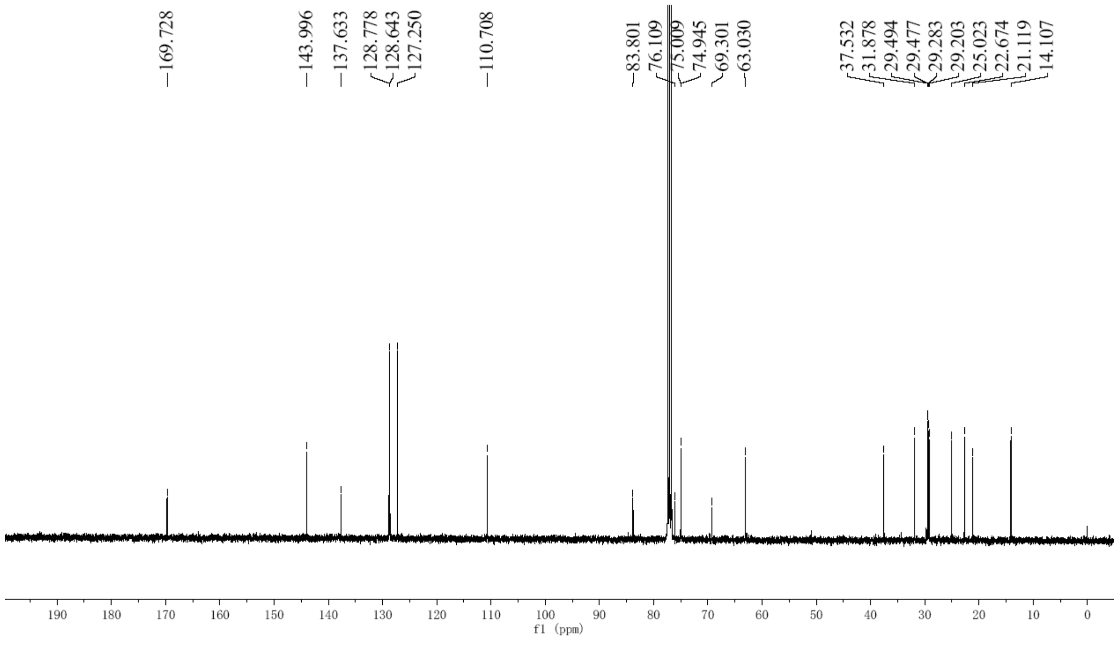

Figure S20. ^{13}C-NMR spectra of compound **7d**.

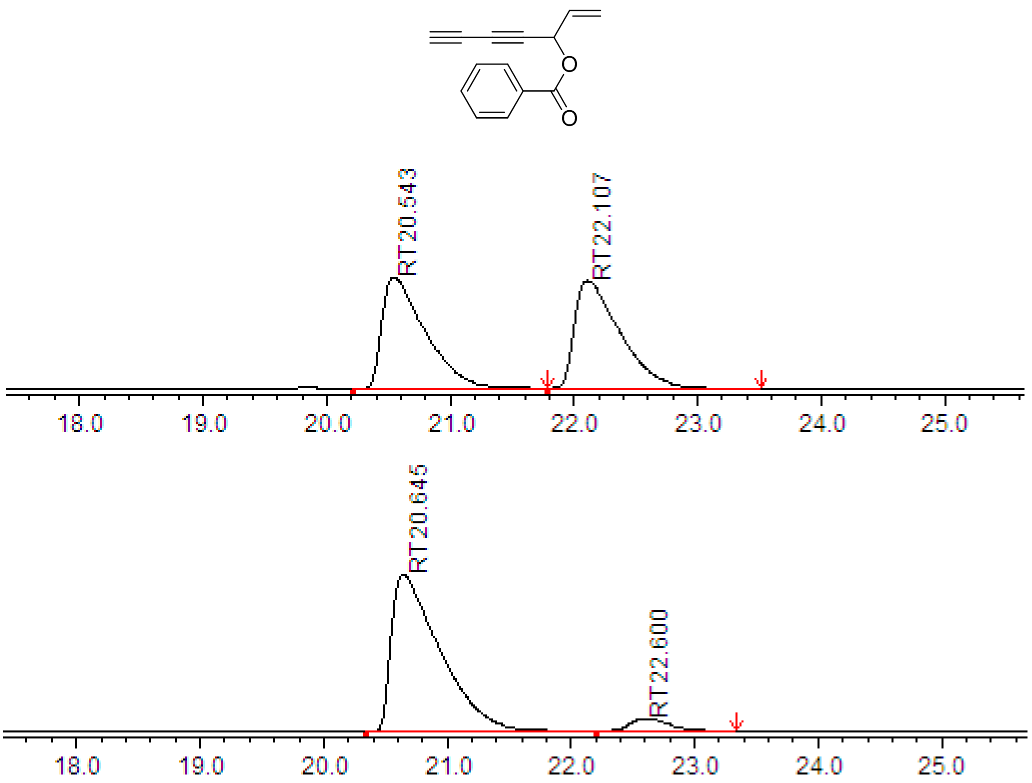

Figure S21. HPLC analysis of **6a** and its corresponding racemate.

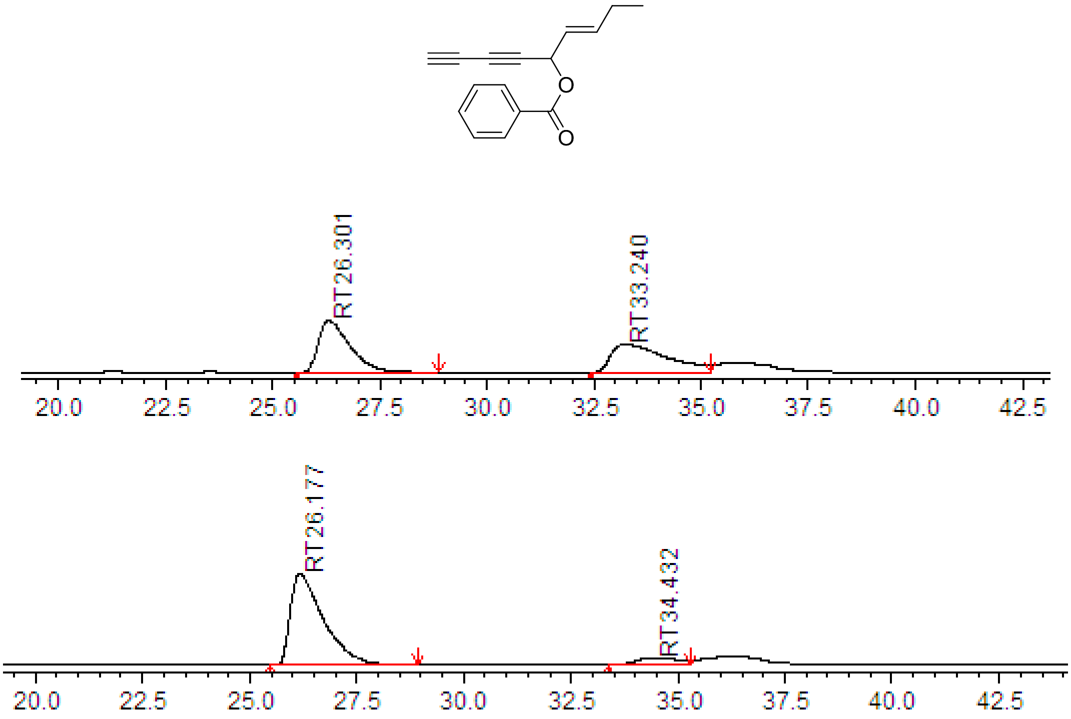

Figure S22. HPLC analysis of **6b** and its corresponding racemate.

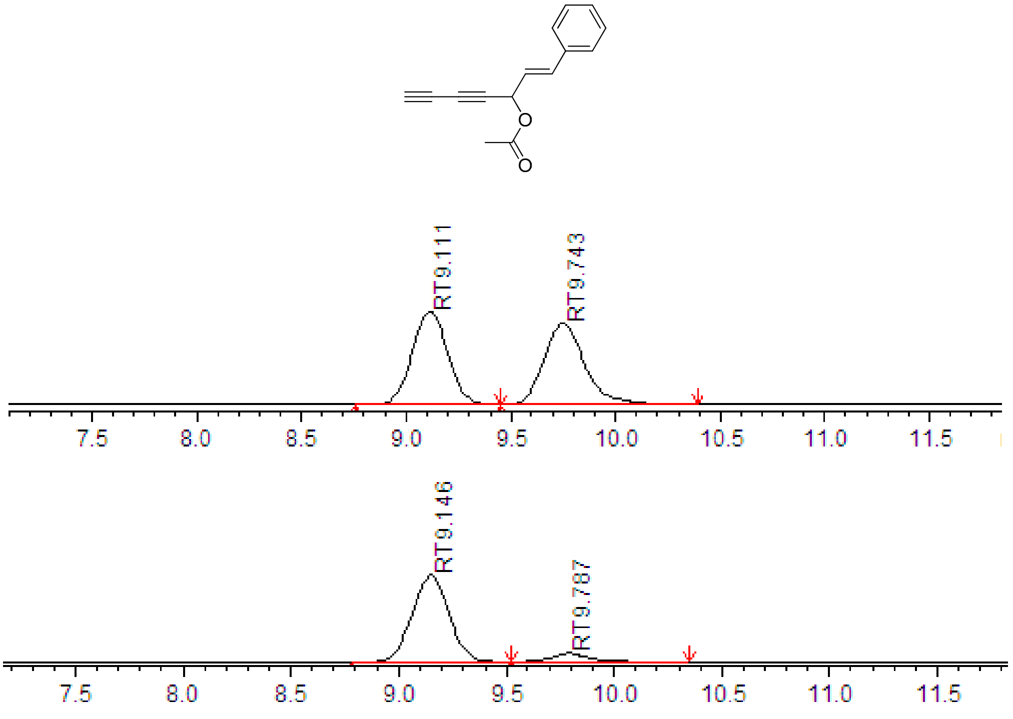

Figure S23. HPLC analysis of **6c** and its corresponding racemate.

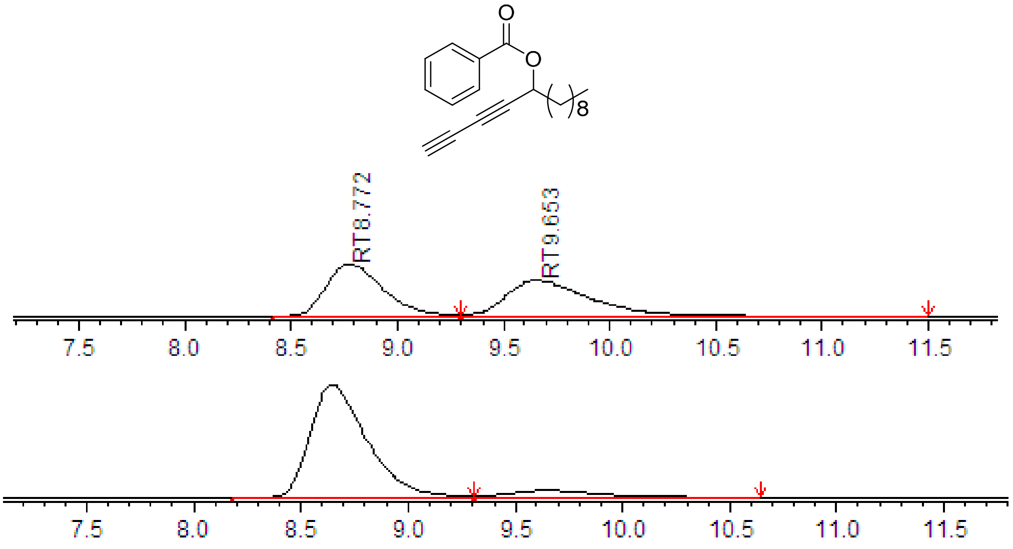

Figure S24. HPLC analysis of **6d** and its corresponding racemate.

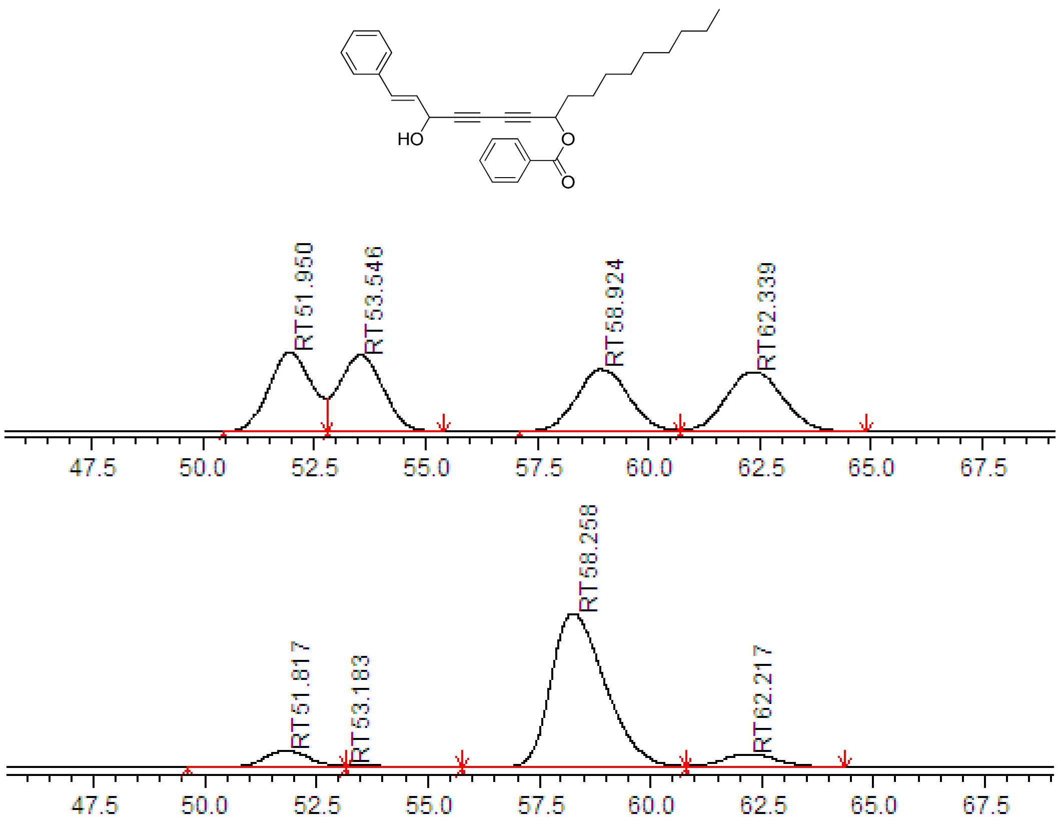

Figure S25. HPLC analysis of **7c** and its corresponding racemate.
